# Intersectional violence and the health of migrant women in Brazil and Brazilian emigrant women abroad: a scoping review

**DOI:** 10.1590/0102-311XEN197625

**Published:** 2026-05-18

**Authors:** Cristiane Batista Andrade, Fernanda Mendes Lages Ribeiro, Corina Helena Figueira Mendes, Rosana Evangelista-Poderoso, Bárbara Santos Leitão, Maria Isabel Ramos da Silva Abelson

**Affiliations:** 1 Escola Nacional de Saúde Pública Sergio Arouca, Fundação Oswaldo Cruz, Rio de Janeiro, Brasil.; 2 Instituto Nacional de Saúde da Mulher, da Criança e do Adolescente, Fundação Oswaldo Cruz, Rio de Janeiro, Brasil.; 3 Faculdade de Ciências Médicas, Universidade Estadual de Campinas, Campinas, Brasil.

**Keywords:** Human Migration, Women, Intersectionality, Violence, Migrant Health, Migración Humana, Mujeres, Interseccionalidad, Violencia, Salud del Migrante

## Abstract

Migratory flows have expanded with the increased movement of women from Latin America and other continents to Brazil, in addition to the emigration of Brazilian women to the Global North. Considering the context of the feminization of migration and the importance of understanding women's oppression in the face of displacement, this scoping review analyzes, from an intersectional perspective, scientific evidence on health and expressions of violence against migrant women in Brazil and Brazilian women abroad. The adopted databases are VHL, Cochrane, Embase, PubMed, SciELO, Scopus, and Web of Science, using descriptors related to the topic. Twenty-six documents were included for analysis. Regarding the health of migrant women, the results indicate that migration has implications for access to and care for health, sexual and reproductive health, mental disorder, and the manifestation of suffering, in addition to barriers to access to health services due to intercultural and linguistic aspects. Both migrant women in Brazil and Brazilian women abroad suffer from intersectional violence, including expressions of racism, xenophobia, racial and gender inequality in the workplace, obstetric, sexual, and patrimonial violence, and conditions analogous to slavery, in addition to the hypersexualization of Brazilian women in the Global North. However, despite the challenges, oppression, and violence, the review shows the resistance and protagonism of migrant women throughout their life trajectories, as individual and collective strategies are developed to guarantee their rights as migrants and women.

## Abstract (es)

Los flujos migratorios se han intensificado, con un aumento del movimiento de mujeres de América Latina y de otros continentes hacia Brasil, así como la emigración de mujeres brasileñas hacia el Norte Global. Considerando la realidad de la feminización de la migración y la importancia de comprender las opresiones vivenciadas por las mujeres en este contexto, esta revisión analiza, desde una perspectiva interseccional, evidencias científicas sobre la salud y las expresiones de violencia contra las mujeres migrantes en Brasil y las mujeres brasileñas en el extranjero. Las bases de datos utilizadas fueron BVS, Cochrane, Embase, PubMed, SciELO, Scopus y Web of Science, con descriptores relacionados con el tema. En total, se incluyeron 26 documentos en el análisis. Respecto a la salud de las migrantes, los resultados indican que la migración impacta en el acceso y en el cuidado de salud, en la salud sexual y reproductiva, en la salud mental y en las manifestaciones de sufrimiento, además de evidenciar barreras de acceso a los servicios de salud asociadas a aspectos interculturales y lingüísticos. Tanto las migrantes en Brasil como las mujeres brasileñas en el extranjero enfrentan violencia interseccional, que incluye racismo, xenofobia, desigualdades raciales y de género en el entorno laboral, violencia obstétrica, sexual y patrimonial, condiciones análogas a la esclavitud y la hipersexualización de las mujeres brasileñas en el Norte Global. Pese a los desafíos, a la opresión y a la violencia, la revisión también destaca las resistencias y los protagonismos de las migrantes a lo largo de sus trayectorias, expresados en el desarrollo de estrategias individuales y colectivas para garantizar sus derechos como migrantes y mujeres.

## Introduction 

Migratory flows have expanded in Latin America, especially in Brazil. Official Brazilian data indicate that 481,000 residency applications were granted in the country from 2022 to July 2024 and that, although men are the majority, the number of women, children, and adolescents has grown significantly, with the most prevalent nationalities being Venezuelan, Bolivian, Colombian, and Argentinean ^1^. In this same period, approximately 139,200 people requested asylum in the country, with prevalence of Venezuelans, but also including Cubans and Angolans: “*...96.0% of them were Venezuelans, with women representing 47.2% of recognized refugees*” ^1^.

It is extremely necessary to conduct studies and produce data (empirical, theoretical, and methodological data) on the migration of women, especially regarding their motivations, since they have historically been seen as the support for their companions, and not as protagonists of their own story ^2^. 

It is increasingly common for women to migrate alone or with their children and they tend to seek employment to support their families ^2^. Therefore, in Brazil, as a country that has received a diverse range of migrants, especially in recent decades, we should understand the intersectional violence ^3^ against migrant women for provision of health care, prevention of violence, and development of effective policies to address this serious public health issue.

Women protagonism and challenges experienced in human displacement have been increasingly discussed from a feminist perspective, elucidating the growing movement and the diverse reasons for migration ^4^. While emigration is motivated by deficient rights, with women facing limited social mobility policies geared toward improving their living conditions, which contributes to their increased vulnerability ^5^.

Furthermore, in migratory contexts, violence can be exacerbated, showing how migration is intertwined with colonialism and body subjugation ^4^. Historically, violence against women is associated with male domination and exacerbated by colonization, in which the racialization and sexualization of gender relations characterize the structures that have been built since the European invasion of Latin America. Within the colonial gender paradigm, cruelty, impunity, and the naturalization of violence against women are increasingly prevalent, affecting their lives, including cases of femicide ^6^.

In this sense, intersectional studies inform analyses of oppression and violence against women, such as sexism, racism, and classism, complicating the dynamics of violence. Therefore, we address the diversity among women, even among those of the same social class and ethnicity. In other words, analyzing how the intersections of oppression shape their experiences is beneficial for developing public policies to meet their needs and address rights violations and violence ^5^ ^,^ ^7^. 

As for migration processes, there is a consensus that they impact women’s lives differently, depending on their nationality, immigration status, sexual orientation, gender identity, race/ethnicity, and social class ^5^ ^,^ ^8^ ^,^ ^9^. Consequently, adopting intersectionality as an axis of analysis provides a more in-depth insight into the experiences of migrant women, shedding light on their oppressions and resistance beyond asymmetrical gender relations, also including distinctions and discrimination based on race and social class, access to employment and migration conditions, affinity with the destination country’s language, among other indicators ^8^ ^,^ ^9^.

Thus, considering that violence is intersectional and complex, Collins ^3^ reiterates that it is a social problem that causes suffering and harm to affected individuals. The author believes that, beyond the intersections of racial, gender, nationality, and social class violence, it is crucial to identify its determinants within the context of exploitation in a capitalist society, especially in countries that underwent colonization ^3^, a hallmark of Latin America. Therefore, if we consider these reflections on migrant women, we can infer that the more impoverished, the black, the indigenous, and those from the Global South are the most affected by racial and gender-based violence − a perspective adopted in this article.

If women’s movements involve countless challenges, how do migration processes and their health interrelate? A recent literature review ^10^ found that, in addition to migratory difficulties, women also face exacerbated vulnerabilities and social and economic inequalities, weak employment relationships, and the failure of services to meet their health needs, especially during the pandemic. Women face barriers in reconciling productive work with family care, which is expressed by the overload of demands, impacting their physical and mental health. They are often subjected to racial, gender-based, and xenophobic violence, which can cause or aggravate psychological distress and disorders ^10^. 

However, this violence is not limited to Brazil, being also experienced by Brazilian women abroad; after migrating to the Global North, they report suffering racism and xenophobia, in addition to hypersexualization of their bodies, stigmatized as “easy” and sensual ^11^ ^,^ ^12^, and obstetric violence ^11^. Therefore, elucidating how our compatriots experience migratory processes and access to health care in the Global North is important to unveil the violence ^11^ and the colonial gender pattern that is perpetuated ^6^. This perspective reinforces the Brazilian Migration Law (*Law n. 13,445/2017*) ^13^, which fosters studies and research on Brazilian women abroad, as this population is a responsibility of the Brazilian State.

Thus, this scoping review aims to analyze scientific evidence on the health and expressions of violence against migrant women in Brazil and Brazilian women abroad. The research question is: What is the scientific evidence on the health and expressions of violence against migrant women in Brazil and Brazilian women abroad?

## Methods

This scoping review aims to present a broader view of a given topic and discuss the diversity of knowledge, identifying gaps and supporting future systematic reviews ^13^. We followed the PRISMA-ScR (*Reporting Items for Systematic Reviews and Meta-Analyses: Extension for Scoping Reviews*) recommendations ^14^ ^,^ ^15^, which require registering a scoping review protocol, which was performed in October 2024 (https://osf.io/c9vba/overview), and the databases and descriptors used can be consulted. The strategy was developed by a librarian with a PhD in health sciences, specialized in the main health and interdisciplinary databases.

After establishing the research objective and question, we defined the population, concept and context (PCC) criteria of the review ^14^, namely: (a) Population: migrant women (young women aged over 18 years, female adults and older adult women); (b) Concepts: violence and health care; and (c) Contexts: Brazil as a destination for transnational migrant women and other countries as a destination for Brazilian emigrant women. 

Box 1 presents the databases consulted, namely SciELO, Virtual Health Library (VHL), Embase, Web of Science, Scopus, Cochrane, and PubMed, the fields used, and the number of documents found.

**Box 1:** Databases consulted, fields used, and number of documents.

DATABASES ADOPTED	FIELDS USED	DOCUMENTS
VHL	Title, subject, and abstract	137
Cochrane	Title, subject, abstract, and MeSH descriptor	4
Embase	All fields, EMTREE	67
PubMed	Title/Abstracts, MeSH terms, all fields	443
SciELO	All indexes	71
Scopus	Title, subject, and abstract	45
Web of Science	TS = topic and AB = abstract	269
Total with duplicates		1,036
Total after automatic duplicate exclusion via Zotero		899
Total after manual duplicate exclusion on Rayyan platform		895
Total without duplicates		895

Source: prepared by the authors.

The documents retrieved from the databases (n = 1,036) were entered into the Zotero reference management software (https://www.zotero.org/) for duplicate detection and removal, totaling n = 899 after deletion. This total was then entered into the Rayyan system (https://www.rayyan.ai/), and the duplicate detection function was activated once again. This identified four additional duplicate documents, resulting in a final sample of n = 895. In this same program, the double-masked selection procedure was performed by two independent researchers, eliminating the need for a third party, as conflicts were resolved by consensus.

**Figure 1:**
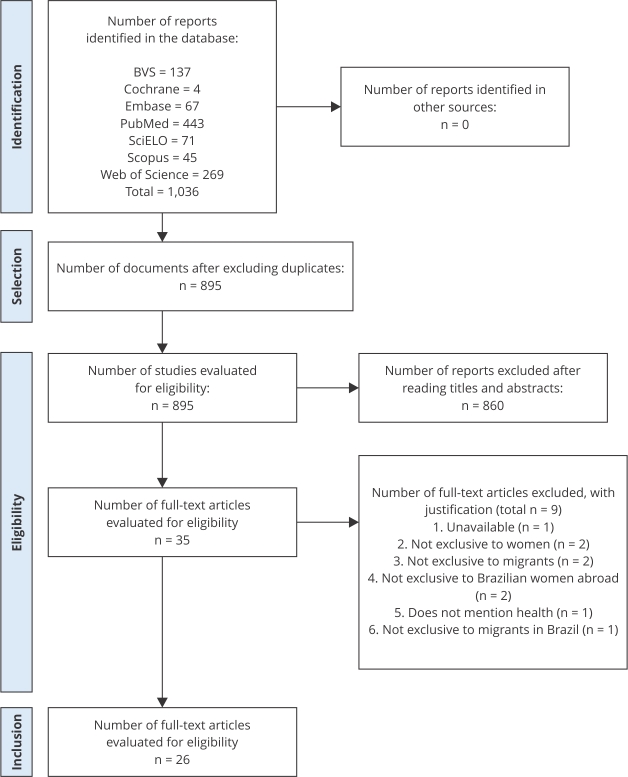
PRISMA flowchart for selecting documents for scope review.

The inclusion criteria were articles, books, theses, and dissertations: (a) addressing health and violence against migrant women (young women aged over 18, adult women, and older adult women) in Brazil and Brazilian emigrant women abroad; (b) available online and in full; and (c) in Portuguese, English, or Spanish. The exclusion criteria were documents: (a) addressing topics outside the scope of the research; (b) addressing men, women, children, or adolescents; and (c) unavailable in full. Figure 1 Prisma ^15^ below presents the methodological approaches of the review.

## Results 

After defining the inclusion of articles for analysis, we created Box 2 with the main results of the research on the expressions of violence and the health of migrants.

**Box 2:** Summary of selected articles: nationality of migrants, location in Brazil and abroad, objective, methodology, main results on health and expressions of violence.

STUDY (YEAR)	POPULATION, CONTEXT, AND PROFILE OF PARTICIPANTS AND LOCATION IN BRAZIL (PCC)	OBJECTIVE	METHODS	CONCEPT MAIN HEALTH RESULTS	CONCEPT MAIN RESULTS ON EXPRESSIONS OF VIOLENCE
TRANSNATIONAL MIGRANTS IN BRAZIL					
1. Meneghel et al. ^16^ (2022)	Women of different nationalities on the Brazilian borders	To analyze the deaths of women due to abuse in the 122 municipalities on the Brazilian border, and to measure and identify associated factors	Quantitative - data from the SIM (acronym in Portuguese). Independent. variables: migration, income concentration, ethnicity/skin color, population, religion, and sexual violence	The study delved little into the discussion of health. However, it shows that indigenous women, in addition to suffering from violence (13% of 181 femicides), face serious health problems. It reiterates that migration processes influence the health of populations	Border regions can be affected by drug, arms, and human trafficking, leading to contemporary slave labor, sexual exploitation, forced prostitution, femicide, and homicide. Some 1,384 female deaths were recorded from 2000 to 2015, with an average rate of 5.8/100,000. The distribution pattern of these deaths was predominantly concentrated in the central arc (Rondônia, Mato Grosso, and Mato Grosso do Sul), with the largest contingents of migrants and reports of sexual violence, especially in larger and more populous municipalities. There has been an increase in small municipalities. There is underreporting of cases, especially among indigenous women
2. Brazilian Ministry of Health ^34^ (2013)	Health care professionals, social workers and other sectors involved in migration and violence against women	To compose the teaching material for the “women migration and trafficking for sexual exploitation and degrading labor” course to promote the training of professionals in health care, social care and other governmental and non-governmental sectors	Qualitative - data from the literature, the SIM, and other health databases	Migrant women’s health is affected by unsanitary conditions, lack of access to health care services, and exposure to occupational hazards and diseases. This highlights the responsibility of health care services in identifying and intervening in situations of violence against women	Human trafficking affects poor, unemployed and young women, who are the most vulnerable to forced labor and economic or sexual exploitation. They are more exposed to migration between Brazilian states or to other countries, making them vulnerable in situations of violence, commercial sexual exploitation when it comes to adolescents or children, and forced prostitution when they are adults, with or without minimum payment for their work
3. Brazilian Ministry of Health ^35^ (2013)	Health care professionals, social workers and other sectors involved in migration and violence against women	To compose the teaching material for the “women migration and trafficking for sexual exploitation and degrading labor” course to promote the training of professionals in health care, social care services, and other government and non-governmental sectors. The book contains 11 articles on the topic and addresses Brazilian women abroad	Qualitative - data from the literature and the experience of the Suindara project, for professional training in the SUS (acronym in Portuguese)	The impacts of violence on health include sexual exploitation, degrading work, difficulties in sexual and reproductive health care, psychological distress and mental disorders, chemical dependency, substandard living and health conditions, mistreatment, human trafficking in rural and urban areas, material vulnerability, and the transfer of people to places without emotional, community, and social connections. The rupture of social, emotional and symbolic relationships, difficulties in cultural integration, isolation, loneliness and the pressures and tensions of everyday life, underlying the migration process and the situation of irregularity, can lead to states of psychological fragility, leading to psychological distress and mental disorders	Migrant women are among the most vulnerable to sexual violence in the workplace, and black women are the main victims of human trafficking, especially in the Brazilian border region. Women experience institutional violence in the health care and social care sectors and experience gender-based, sexual, physical, psychological, collective, and xenophobic violence
4. Oliveira ^36^ (2022)	Bolivian, Haitian, Angolan, Congolese and Venezuelan women in São Paulo (Brazil)	To discuss the migration processes and mental health of migrant and refugee women in São Paulo	Qualitative - questionnaire and interviews with nine migrants	Psychosocial distress, situations of vulnerability, and oppression based on gender, race, and social class indicate adverse effects on migrants’ mental health. Women have exhausting routines, with few leisure moments. Domestic chores and family care are associated with this overload. They face discrimination in health care services because they are migrants; there is distress related to family breakdowns, with sadness, fear, and concerns about family subsistence deteriorated by COVID-19. Anxiety attacks, sleep disorders, headaches, panic attacks, and the use of psychotropics are reported	Racism, discrimination, including in health care services, xenophobia, moral harassment, hunger and political persecution in the country of origin, and verbal abuse affect women
5. Avellaneda Yajahuanca ^37^ (2015)	Bolivian women in São Paulo	To analyze the experiences of Bolivian women during health care, childbirth, and postpartum care	Qualitative - participant observation and individual interviews	Prenatal and postpartum health care are precarious, along with severe language and cultural barriers. Due to their work in workshops, they sleep poorly, the work setting mirrors their home, the food is poor, there is poor ventilation, and fabric dust affects respiratory health. Musculoskeletal disorders are related to sewing and fatigue. Workshops are precarious, with electrical problems, water leaks, and mold. Regarding childbirth, they feel safer at home. There are deficiencies in care, with disregard for cultural dimensions during prenatal and postpartum care	Obstetric violence (kristeller maneuver, episiotomy, use of forceps; repeated vaginal examinations; difficulties in interacting with health care professionals due to cultural and linguistic barriers; institutional violence with xenophobic expressions by professionals; racism); domestic violence; lack of maternity leave; financial violence (robberies) in the workshops; exhausting work hours, lack of pay, cases of contemporary slave labor, and lack of labor rights have been reported. There are deportation threats from their employers. Their children suffer from bullying/xenophobia
6. Balestro & Pereira ^17^ (2019)	Gambian woman in Rio de Janeiro (Brazil)	To discuss language and culture in the context of feminized migration	Qualitative - audiovisual resources available online (interviews) with a refugee, in addition to the use of official data on refugee status	The respondent describes her motivation to leave her country as a result of her desire to study, have a profession, and be independent. There are language difficulties in Brazil and a need to overcome barriers to access to education, health care, and other public services, especially in migrant entry points	The respondent addresses the culture of child marriage in her country, as well as patriarchal relations that reverberate in gender inequalities, in addition to forced migration and xenophobia
7. Calderón Uribe et al. ^18^ (2021)	Brazilian women in the context of internal displacement (Porto Alegre, Rio Grande do Sul State, Brazil)	To understand the perspectives of internally displaced Brazilian women, who are family breadwinners, and the state’s actions in the face of this migration	Qualitative - case study with discourse analysis of three interviews with women who experienced displacement in Porto Alegre	Forced displacement, substandard living conditions, structural violence, and the lack of effective public policies that address women’s health needs have direct implications for their physical and mental health and that of their families. The lack of an integrated and accessible health care system for this population is one of the main deficiencies observed in the context of social care in response to displacement. One case of a homeless woman was identified	The women interviewed and their families faced several forms of violence during their displacement, in addition to forced migration due to environmental issues. This violence can be manifested physically, directly, indirectly, and symbolically. Their experiences and narratives are devalued and become a form of violence perpetuated by negligence and lack of recognition on the part of the state. Persistent insecurity, exclusionary urbanization, and substandard living conditions are seen as consequences of structural violence that perpetuates poverty and exacerbates vulnerability to further displacement. There are reports of domestic violence and the death of children and partners due to drug trafficking
8. Arruda-Barbosa et al. ^19^ (2024)	Venezuelan women in the State of Roraima (Brazil)	To analyze the violence perpetrated against Venezuelan immigrant sex workers using an intersectional analysis	Qualitative - interviews with Venezuelan female sex workers and online media reports on the topic	Precarious health conditions arise from social and economic vulnerability. Violence and sexual exploitation also cause psychological distress and mental illness, triggering depression and anxiety. Offering additional payment for unprotected sex increases exposure to sexually transmitted infections (STIs) and other health problems. Women’s health directly reflects the condition of migration associated with gender and racial inequalities, highlighting the need for health care and the interfaces with these oppressions	Structural violence with unemployment, poverty, hunger, and the need for subsistence occurring to Venezuelan women, with physical violence being the most feared, as they are attacked, including the use of bladed weapons by clients, partners, and even third parties. There is gender-based and sexual violence (rape), psychological violence, verbal violence, and sexual exploitation, all exacerbated by forced migration, poverty, and oppression related to race/ethnicity; patrimonial violence (theft of money), and discrimination against low wages due to the fact that they are Venezuelan
9. Fernandes & Onuma ^20^ (2024)	Women of different nationalities in Brazil	To identify violence for the implementation of public policies	Qualitative - bibliographic survey, documentary analysis and official data from the National Committee for Refugees (CONARE) on requests for refuge in Brazil	Situations of post-traumatic stress due to forced migration and adaptation to the new country, aggravated by socioeconomic vulnerability, are challenges and impacts on the mental health of refugees. Barriers to accessing health care are faced by many women in refugee situations, reflecting the neglect of Brazilian statistical data, in which these women are often addressed as a homogeneous group, negating the need for consideration of their specificities and plural situations, as well as different possible intersections of oppression	There are reports of discrimination, forced migration, and sexual, cultural, and psychological violence; violence related to motherhood, discrimination, and lack of access to health care services, affecting physical and emotional health; separation of family members due to the lack of infrastructure in destination countries, in addition to difficulties with motherhood (pregnancy, postpartum period and care for children/family members); Intersecting discrimination based on social class, gender (gender identity and sexual orientation), race/ethnicity, ableism, religious intolerance and ageism (against elderly migrant women), and lack of guarantees of basic rights, intensifying the economic, social, political and cultural vulnerabilities of these women
10. Ferreira et al. ^21^ (2022)	Colombian, Haitian, Venezuelan and Uruguayan migrant women in Foz do Iguaçu (Paraná State, Brazil)	To understand the psychological impacts of migration on the lives of female university migrants	Qualitative - descriptive and exploratory. Interviews with semi-structured questionnaires and an intercultural sociodemographic form and content analysis	The psychological health of women who migrate for education is directly impacted, from the migration process to their time at university: psychological distress, depressive and anxious states, somatization, sadness, low self-esteem, and weight gain. Transnational motherhood also exacerbates psychological distress (fear, anxiety, loneliness). Universities need to commit to gender equity, acceptance, and retention policies, based on an intersectional analysis. The study also notes the resilience and autonomy of women who migrate	Reports of discrimination related to the masculinized space of some university courses; situations of gender and sexual violence from the migration process to their time inside and outside the university; structural violence, abuse and micro abuse related to race, gender, ethnicity, nationality, culture, and language, among others; forced migration among venezuelan women, exploitation at work with low wages and intense working hours (14 hours/day) and precarious housing, and racism against a haitian woman who discovers she is black in brazil, reporting much distress, and against a colombian woman who has indigenous ancestry
11. Makuch et al. ^22^ (2021)	Venezuelan women in the State of Roraima	To analyze experiences of violence against Venezuelan women in shelters in the State of Roraima	Qualitative with focus group	Faced with the violence they suffered, the women reported fear of their partners and of being abandoned by them, as well as feelings of guilt. They also expressed fear of other men living in the shelter, particularly as to using restrooms	Reports of domestic violence (physical and verbal abuse and psychological threats), sexism, and xenophobia outside of shelters; violence perpetrated against children by partners, committed by women who assaulted their partners and other women who had romantic relationships with them; and patrimonial, physical, and verbal violence perpetrated by Venezuelans in shelters. Some are aware of the Maria da Penha Law and use it as a self-protection strategy
12. Santos ^38^ (2019)	Congolese women in Rio de Janeiro	To reflect on the refugee process and mental health of Congolese women	Qualitative - participant observation and interviews with Congolese refugees	Women experience social distress and poor living conditions, depression, anxiety, loneliness, and sadness. There is a close relation between their experiences as refugees and unemployment, language difficulties, and loneliness, with cases of emotional exhaustion, distress, headaches, insomnia, stress, tachycardia, fatigue, grief, depressive and psychosomatic symptoms exacerbated by armed violence in the *favelas* where they live. Forced migration and political persecution can cause trauma, fear, suffering, family separation, discouragement, anguish, and stress. There are reports of slow health care. Faith and spirituality are resources for support in the face of challenges	Reports include domestic violence; armed/urban violence with feelings of fear, anguish, and crying when faced with assaults and armed individuals; forced migration; armed confrontations in the homeland; political persecution; xenophobia and racial division at work; sexual violence in armed confrontations in the homeland; racism, including in public health care services, and xenophobia suffered by their children in Brazilian schools
13. Pinto & Amaral ^23^ (2016)	Cuban women	To analyze texts and reports about the mobility of Cuban female doctors in Brazil	Qualitative - documentary analysis on government websites, commercial and non-commercial media on migration (from 2003 to 2013)	Although the article does not address the health of Cuban female doctors, it indirectly addresses the work of these health care professionals	Reports of racism by Brazilians when questioning whether the doctors were really doctors, as expressed in the media: “these Cuban doctors look like maids”, or “are they really doctors?”
14. Santos et al. ^24^ (2015)	Bolivian women in São Paulo	To identify the characteristics of Bolivian women who are pregnant as a result of rape	Qualitative - retrospective study, and quantitative - documentary study with 38 Bolivian women in a public legal abortion service who were victims of sexual violence	Lack of awareness of public health care services, informal employment relationships, and irregular migration ultimately preclude women from accessing health care services. The authors indicate underreported sexual violence cases, as well as difficulties in seeking legal abortion. Most women arrived at the hospital after being referred by the police and other health care institutions	Occurrence of sexual violence (rape; 63.2% by strangers), physical violence and threats
15. Serrano & Martin ^25^ (2022)	Bolivian women in São Paulo	To analyze domestic violence against Bolivian women workers in home-based sewing workshops	Qualitative - ethnography, interviews, participant observation and focus group	Women are prevented by their employers from seeking health care services; they have little access to adequate food, and they are denied maternity leave. Regarding access to services, they face linguistic and cultural obstacles, such as representations of the health and care process that differ from those of Brazilian society. When faced with domestic violence, they reported suffering from depression, stomach pain, shame, fear, stress, and high blood pressure	Bolivian women experience precarious working conditions: working days of more than 12 hours, little rest, and extremely low wages, bordering on slavery-like and unhealthy conditions, and they experience racism, classism, sexism, and xenophobia. Regarding domestic violence, because they are undocumented, they suffer from language barriers, and being far from their home country and without a support network makes it difficult for them to seek health care services. Some report death threats and financial violence from their partners
16. Supimpa et al. ^26^ (2023)	Venezuelan, Haitian and Tunisian women in the city of Curitiba (Paraná State)	To describe the experiences of migrants during labor and birth in two public maternity hospitals in Curitiba	Qualitative - hybrid thematic oral history and semi-structured interviews	During the labor and birth process, the migrants reported pain, fear, loneliness, sadness, fear of cesarean section, and distrust of the health care team. There were also reports of trust in the teams, happiness, and a feeling of having been well cared for by nursing staff (newborn care, breastfeeding, hygiene, etc.). Difficulties in communication between health care professionals and migrants were also noted, showing the need for sensitive cross-cultural health care	Unwanted vaginal touches during childbirth and obstetric violence
17. Silva & Justo ^27^ (2020)	Brazilian internal migrants (states of São Paulo and Paraná)	To investigate the mobility of women who move from city to city in the inland region of the State of Mato Grosso do Sul	Qualitative - cartography with interviews with two women	Homeless people are often viewed socially as sick, far from the ideal of a nuclear family. There are reports of childhood anxiety and crying when recalling situations of violence, and there are reports of use of legal and illegal drugs and medication	Women migrate to escape domestic gender-based violence and to seek better living and working conditions. One respondent reported experiencing physical and emotional violence as a child due to her homosexuality
18. Souza et al. ^28^ (2020)	Haitian women in several Brazilian cities	To understand the repercussions of COVID-19 and the social determinants of health	Qualitative - participatory action with Haitians. Snowball sampling	Fear COVID-19, scarce financial resources, the impact of racism and prejudice on mental health, difficulties and overwhelm in balancing work and family care during the pandemic, as daycare centers and schools were closed, unemployment, anxiety, worry, stress, crying, and lack of sleep were reported. Health care services and social support strengthen pandemic coping strategies	There is prejudice, racism and discrimination in the job market because they are women, as they receive lower salaries compared to men
19. Gehlen et al. ^29^ (2023)	Venezuelan women in Rio Grande do Sul State (Brazil)	To analyze the vulnerabilities of Venezuelan women and their experiences of violence in refugee status	Qualitative - interviews with Venezuelans living in a city in Rio Grande do Sul	Mental health deteriorated (panic disorder, depression, feelings of sadness, shame, humiliation, and inferiority) and illnesses worsened with migration; family relationships (with relatives and/or partners) were disrupted, with little or no support network (family or government). They reported satisfaction with SUS services; however, they felt that health care professionals had difficulty serving them	Xenophobia, hunger, domestic violence, racism, sexism, sexual violence, stigmatization, classism
20. Hora ^39^ ^)^ (2023)	Congolese women in Rio de Janeiro	To analyze access to health care for Congolese women in Rio de Janeiro	Qualitative - interviews with FHS managers and professionals and Congolese women	Congolese women say that health care at family clinics is good, but they identify a lack of support within the SUS (precariousness of health care work and underfunding of public policies within the SUS). There is a lack of intercultural mediation, delays in obtaining health care, poor medical care and demands for family planning and prenatal care. There is no production of systematized data on the health of migrants and there is a need for professional training (permanent health education) in the SUS, and there is production of educational materials on health for migrants	Violence against migrant women is a challenge, as is the case with sexual violence, and requires monitoring by mental health care services. There is obstetric violence, institutional racism, urban and armed violence in Rio de Janeiro (shootings and police operations in the territories where they live), and experiences of forced migration
BRAZILIAN WOMEN ABROAD					
21. Barata ^11^ (2022)	Brazilian women in Portugal	To discuss experiences of racism and obstetric violence against brazilian women in Portugal	Qualitative - interviews with three Afro-brazilian women in Portugal, participant observation, focus groups and participatory artistic creation on obstetric care	The fact that women report experiencing obstetric violence, xenophobic violence, and racial violence has implications for sexual and reproductive health, self-esteem, and quality of life, especially during pregnancy and childbirth. One woman reported postpartum depression associated with obstetric violence. Barriers to accessing health care services are also reported	Gender-based/obstetric violence, the kristeller maneuver, verbal and physical abuse, mistreatment, body objectification, non-consensual medical interventions, episiotomies, inappropriate procedures, xenophobia and racism, hypersexualization of brazilian women, and verbal abuse during public breastfeeding were all found, among other incidents. Furthermore, it was commonly heard that women have “bad uteruses” and are of mixed race, which reinforces xenophobia and racism
22. Chedid & Hemais ^30^ (2022)	Brazilian women in the global north	To analyze, through Spivak’s postcolonial theory, how Brazilian women in the tourism context in the global north are subjugated by foreigners	Qualitative − case study through 14 interviews with Brazilian women who traveled for tourism in the global north	The article does not directly address specific health outcomes. However, subordination combined with experiences of physical and psychological violence can have direct impacts on women’s health. Discrimination and objectification in the tourism context can also cause significant emotional distress. Regarding sexual violence, when harassed by men, they feel fearful, upset, threatened, guilty, and unable to react immediately. Reduced autonomy and self-care can impact their ability to seek medical care in foreign settings	The main types of violence reported are psychological and physical violence. Psychological violence is expressed through insults and sexist and xenophobic comments, which produce feelings of inferiority. They are treated as objects of desire, and reinforce fetishized representations of Brazilian nationality. Physical violence involves aggressive approaches, shoving, unwanted touching, and sexual violence, causing immediate harm, in addition to fear and insecurity. Furthermore, they are subject to sexism and xenophobia
23. França & Oliveira ^12^ (2021)	Brazilian women in Portugal	To analyze the resistance expressed on social media by Brazilian women in Portugal against discrimination and prejudice	Qualitative - interview with the coordinator of the social network on instagram “*brasileiras não se calam*” and analysis of the discourse of the posts and netnography, from decolonial and intersectional perspectives	Based on the posts of Brazilian women on the social network analyzed, the feelings were of frustration, sadness and humiliation	Prejudice, discrimination, hypersexualization, sexism, and racism reiterate coloniality, even today, and its impacts on the lives of Brazilian women in the global north, with reports of symbolic, psychological, and moral violence, verbal abuse, harassment, and sexual violence. Brazilian women use social media to publicize the violence against them, highlighting their agency and activism
24. Gomes ^31^ (2013)	Brazilian women in Portugal	To analyze prejudiced and discriminatory discourses about Brazilian women in Portugal	Qualitative - participant observation, analysis of media and tourism discourses, use of questionnaires and interviews	The article does not delve much into health issues. However, it notes that Brazilian women are stigmatized as those who “bring diseases” to the Portuguese, especially sexual ones	We have hypersexualization and discourses about the “colonial body,” which are linked to stigmatization such as being sexually available and being lovers of Portuguese men, moral and sexual harassment, physical and sexual violence, and xenophobia. Brazilian domestic workers suffer harassment, especially sexual harassment, racism, and sexism, in addition to working in precarious jobs
25. Mcllwaine & Evans ^32^ (2023)	Brazilian women in London	To analyze gender-based violence against Brazilian women in London (England)	Quantitative/qualitative - questionnaire, focus group and interviews with Brazilian women and managers of migrant care services	Feelings of shame, humiliation, fear (including deportation if undocumented), and isolation. Women in irregular migration situations had more difficulty accessing public services, such as social care, security, and health care, as well as labor rights. One migrant reported depression due to intimate partner violence	Intimate partner gender-based violence, xenophobia, racism, psychological, physical, and sexual discrimination, and sexual harassment in the workplace. Instances of infrastructural violence, that is, violence perpetrated by services providing care to women experiencing violence. Exploitation of women by society, being outside their country of origin, and structural violence are factors that influence the decision to maintain relationships with intimate partners
26. Pelúcio ^33^ (2023)	Brazilian women in Japan, France, Ireland, Italy, Canada, United States, Senegal, England, Denmark, Norway, and Germany	To analyze the narratives of Brazilian women abroad through the femigrantes br podcast	Qualitative - social media (podcast) analysis from the perspective of Black feminisms	One of the podcasts addressed health and intersectionality; however, the article barely addresses this topic	Racism, colonialities of knowledge and power, machismo, sexism, misogyny, and hypersexualized Brazilian women were reported. Black women see their educational path as one of the strategies for confronting racism’s oppressions

FHS: Family Health Strategy; SIM: Brazilian Mortality Information System; SUS: Brazilian Unified National Health System.

Source: prepared by the authors, 2025.

## General aspects of the selected sample

Twenty-six documents were selected for analysis: (a) 20 articles ^11^ ^,^ ^12^ ^,^ ^16^ ^,^ ^17^ ^,^ ^18^ ^,^ ^19^ ^,^ ^20^ ^,^ ^21^ ^,^ ^22^ ^,^ ^23^ ^,^ ^24^ ^,^ ^25^ ^,^ ^26^ ^,^ ^27^ ^,^ ^28^ ^,^ ^29^ ^,^ ^30^ ^,^ ^31^ ^,^ ^32^ ^,^ ^33^; (b) two books ^34^ ^,^ ^35^, and c) four theses/dissertations ^36^ ^,^ ^37^ ^,^ ^38^ ^,^ ^39^. Twenty documents address migrant women in Brazil, of which four studies address migrants of several nationalities ^20^ ^,^ ^21^ ^,^ ^26^ ^,^ ^36^; three address Bolivian women ^24^ ^,^ ^25^ ^,^ ^37^; three address Venezuelan women ^19^ ^,^ ^22^ ^,^ ^29^; two address Congolese women ^38^ ^,^ ^39^; one addresses a migrant from Gambia ^17^; one addresses Cuban doctors ^23^; one addresses Haitian women ^28^; and two address Brazilian internal migrants ^18^ ^,^ ^27^.

Regarding the training of health care professionals or health care service for migrants in Brazil, we found two official documents prepared by the Brazilian Ministry of Health in 2012 ^34^ ^,^ ^35^. We found six articles on Brazilian women abroad, as follows: (a) three about Brazilian women in Portugal ^11^ ^,^ ^12^ ^,^ ^31^; (b) two about Brazilian women in several countries ^30^ ^,^ ^33^, and (c) one about Brazilian women in England ^32^.

Most studies are qualitative, using interviews, focus groups, and analysis of literature and social media. Only one study is quantitative ^16^, and two present a mixed-methods approach ^24^ ^,^ ^32^. Among the regions of the country where the migrants cited in the research reside, we identified: (a) four productions ^24^ ^,^ ^25^ ^,^ ^36^ ^,^ ^37^ in the city of São Paulo; (b) three in the city of Rio de Janeiro ^17^ ^,^ ^38^ ^,^ ^39^; (c) two in the State of Rio Grande do Sul ^18^ ^,^ ^29^; (d) two in the State of Roraima ^19^ ^,^ ^22^; (e) two in the State of Paraná ^21^ ^,^ ^26^; and (f) one in the states of São Paulo and Paraná ^27^. As no time frame was established, we observed that 18 documents were published (five about Brazilian women abroad) from 2020 to 2024, while eight productions were published from 2012 to 2019, indicating the growth of the topic of violence against migrant women in Brazil and Brazilian women abroad in the last four years.

## Health and migration: migrant women in Brazil and Brazilian emigrant women abroad

This section provides a summary of possible inferences from the sample as to the relation between health and migration, with the following categories being addressed by the group: mental health, implications of forced migration and working conditions for health, impacts of violence on health, sexual and reproductive health, and barriers to access to health care services.

Regarding the health of migrant women in Brazil, we observed that they are heterogeneous and are strictly related to nationality, race/ethnicity, generation, social class, sexual orientation, length of stay in the country, and proficiency in the Portuguese language, profession, and labor relations (formal or informal work), in addition to migratory status. In this sense, based on the idea of ​​the plurality of women and their living conditions, we consider that: “*Different peculiarities make them unique and, therefore, demand specific perspectives, both for academic analyses and for their possible consequences in terms of developing public policies related to them*” ^24^ (p. 3).

Migratory processes can involve difficulties in adaptation, social integration, linguistic and social barriers, and decisions that involve having to leave family and children in the country of origin ^36^. Therefore, it was common to find productions that complexify health aspects, associating them to living conditions, migratory processes, and violence against migrants.

We highlight the productions on the mental health of migrants, as the discussions on psychosocial distress and mental disorders are prominent in the sample ^19^ ^,^ ^21^ ^,^ ^34^ ^,^ ^35^ ^,^ ^38^. Evidently, when the study dialogues with intersectionality, mental health appears related to the oppressions related to race, social class, and gender, in addition to the situations of vulnerability of migrants, with their illness expressed by psychosocial distress, anxiety attacks, sleep disorders, headaches, panic syndrome, and the need for psychotropics ^36^.

All changes in living conditions, social and family support networks, difficulties with language and social integration, tensions in daily life, isolation, irregularity in the migration process, and loneliness experienced can have an impact on health, causing distress and the development of mental disorders ^35^ ^,^ ^36^.

Forced displacement exacerbates obstacles to migrants’ mental health maintenance and care: “*When we refer to forced migration, we are referring to individuals who are fleeing war, political, religious, or social persecution, or human rights violations*” ^20^ (p. 4). In refugee situations, women tend to develop post-traumatic stress disorder after leaving their countries, being forced to cope with adaptations, socioeconomic vulnerability, and violence ^20^.

There is social distress, loneliness, anxiety, emotional exhaustion, headaches, insomnia, stress, tachycardia, grieving, anxiety, depression, discouragement, anguish, fears, traumas ^38^, depression, sadness ^29^ ^,^ ^38^, panic syndrome, and feelings of shame, humiliation, and inferiority ^29^. Forced migration and political persecution against Congolese women exacerbate mental distress and disorders, reinforcing the need for mental health care ^39^. 

Arruda-Barbosa et al. ^19^ indicate that, in the case of Venezuelan women, the conditions of forced migration, intersected with racial and gender oppression, exacerbate mental disorder and vulnerability to sexually transmitted infections (STIs), primarily when they are engaged in sex work. In some cases, men even offer higher pay for unprotected sex. Furthermore, experiencing violence is linked to developing depression and anxiety. 

Forced migration is also experienced by Brazilian women who move internally in pursuit of better living and working conditions, which, combined with structural violence, substandard living conditions, and State negligence, has implications for their physical and mental health, requiring the provision of specific and accessible health care ^18^. Even when migrants move voluntarily for reasons such as education within Brazil, that is, outside the context of forced migration, they may experience psychological distress, depression, somatization, sadness, low self-esteem, and weight gain, especially if they become mothers, as this can aggravate feelings of fear and loneliness and be related to anxiety disorders ^21^.

In this review, the migration process is closely related to the health of migrant women, with a significant presence of psychological distress associated with the conditions of migration and life at the destination ^16^ ^,^ ^34^. Depending on their migration status, such as in cases of undocumented status, they face even greater barriers to accessing health care, social care, and other services, aggravated by the lack of social and family support ^24^ ^,^ ^35^. 

The health of migrant women is also interrelated with precarious working conditions, exposure to occupational hazards, unhealthy conditions, workplace violence, and human trafficking for contemporary slave labor and/or sexual exploitation. Depending on their location, whether in rural or urban settings, it is even harder to break free from the human trafficking network, which increases psychological vulnerability and illness ^35^. It is also common for these women to be unemployed or in the informal sector ^36^, which further undermines access to care networks.

Migrants report that their routines are exhausting, with an overload of domestic and family care work, preventing them from having leisure time ^36^. Furthermore, with the advent of the COVID-19 pandemic, Haitian women have spoken about the hardships of balancing family care with work activities, as several schools and daycare centers remained closed, which is closely related to the overload of care work ^28^.

Congolese women also report difficulties in obtaining daycare places for their children and the burden of the sexual division of care. In addition to being responsible for caregiving, in some cases, they report that they are “at the service” of their partners, in accordance with Christian biblical teachings. At the same time, they consider economic subsistence to be a man’s obligation, although they wish to find a job ^38^. Still, regarding reproductive labor, Venezuelan women are responsible for family care and feel obligated to perform it ^22^.

For those who are employed, violence and rights violations are no less prevalent. Bolivian women, for example, are prevented from taking maternity leave and accessing health care services by their employers, as any time away from the machines represents less profit for capitalism. They are also far from support networks and family, making it difficult for them to escape exploitation and violence. Their working conditions often border on slavery, with long, intense workdays and paltry wages ^25^. 

Once they are in sewing workshops, they may find substandard working conditions, such as working in poorly ventilated spaces, mold, and electrical problems (often the same as their housing), triggering respiratory and sleep problems, as well as musculoskeletal disorders due to non-ergonomic machinery and work overload. In addition to using their salaries to support themselves and their children, they also send part of their income to their families back in Bolivia ^37^.

Migration status can also be used as a reason for low wages ^19^, labor exploitation, intense working hours, and poor housing ^21^. This is compounded by the racial division of labor, in which Black women hold positions of low social value, with even lower wages and more precarious activities ^38^. 

Articles about Brazilian women abroad rarely explore health issues, with one of the points raised being the difficulties in accessing health-related services ^11^ ^,^ ^32^. When undocumented, they face difficulty accessing social, health, and security services, as well as labor rights, and fear deportation ^32^.

Fear is a familiar feeling in research on Brazilian women ^12^ ^,^ ^30^ ^,^ ^32^. However, we also observed frustration, humiliation, and sadness ^12^, and shame, humiliation, and guilt ^30^ ^,^ ^32^. If Brazilian women suffer obstetric violence, xenophobia, and racism, they may experience postpartum depression, which impacts self-care and self-esteem ^11^.

Another issue raised by Brazilian women is that they are socially considered to be those who “bring” sexual diseases to foreign men, in this case, Portuguese men, reiterating the stigmatization and hypersexualization of Brazilian women ^31^. This objectification and discrimination can cause emotional stress and negative feelings such as guilt and difficulty in reacting to violence in situations of harassment perpetrated by men ^30^. 

We have repeatedly identified barriers to access to health care services in Brazil as a social factor that further undermines the health of migrant women, who are isolated and deprived of the care to which they are entitled ^35^. Oliveira ^36^ notes the discrimination and xenophobia perpetrated in health care services. The use of xenophobic expressions by health care professionals during childbirth and the postpartum period has also been found ^20^ ^,^ ^37^, as well as cultural and linguistic barriers ^37^ ^,^ ^39^. These situations further preclude women from enjoying their right to health and are reasons for not seeking care in situations of domestic violence ^25^.

Regarding linguistic and cultural approaches to health care, we emphasize the relevance and urgency of providing intercultural mediation programs so migrants can receive quality health care and have their right to health guaranteed ^39^. It should be noted that one of the reasons for migrating to Brazil is the possibility of using the health care services of the Brazilian Unified Nationl Health System (SUS, acronym in Portuguese), such as prenatal and maternity care, appointments with endocrinologists and gynecologists, and vaccinations ^36^. Therefore, ensuring health care that considers intercultural dimensions is essential for adequate provision of health care.

However, publications aimed at training workers in Brazil are based on this recognition to build possibilities of access and care ^34^ ^,^ ^35^, considering the responsibility of public authorities in tackling violence against migrants, notably human trafficking in the forms of slave labor, commercial sexual exploitation, servile marriage and organ trafficking; this perspective is the foundation for the development of training proposals for health care professionals in the SUS network, who must be aware of the vulnerabilities implied in exposure to “*risks of contagion, infections, illnesses, psychological suffering and mental disorders*” ^35^ (p. 35). The significant risk to sexual and reproductive health ^35^, in addition to the difficulty in accessing services that provide this care, is noted and recognized as a fact in the country ^34^. Services need to identify and intervene in situations of violence against migrant women ^34^, recognizing that many of them may suffer institutional violence in health care services ^35^ ^,^ ^36^.

Regarding mental health care strategies for migrant women, we found an important mention of the creation of a specific field of knowledge production and care, called “*‘migrant psychology’, ‘anthropopsychiatry’, ‘transcultural psychology’, or ‘transcultural psychotherapy’*” ^35^ (p. 49), oriented toward understanding the effects of migration on mental health, distress, and illness ^35^.

The “psychology of migration” has focused on the analysis of macrosocial factors, lacking greater consideration of other factors that are closer to the individual, implicated in illness, such as subjective, behavioral, sociocultural, and intersubjective factors ^35^. However, the medicalizing perspective that reduces suffering to the individual and that often guides mental health care practices is criticized.

## Violence and migration: migrant women in Brazil and Brazilian emigrant women abroad

This section presents the types of violence against migrants found by the authors of the sample, including gender-based, racial, xenophobic, and institutional violence. Forced migration is understood here as a violent event that makes women vulnerable by forcing them to move from their places of origin due to direct violence, such as gender-based violence, and indirect violence, such as structural violence, which makes certain territories unsustainable for life, including hunger, the presence of armed groups, and climate disasters, among other factors ^40^. 

We should underscore that, although some documents make specific reference to certain types of violence, a range of productions ^11^ ^,^ ^12^ ^,^ ^19^ ^,^ ^25^ ^,^ ^29^ ^,^ ^31^ ^,^ ^32^ ^,^ ^33^ ^,^ ^36^ ^,^ ^37^ ^,^ ^38^ ^,^ ^39^ intersects violence against migrants, highlighting the complexity of oppression in their daily lives.

The study of Barata ^11^, for example, intersects and racializes obstetric violence, contextualizing the colonial relations that marked Brazil and Portugal. Oliveira ^36^, drawing on Kimberlé Crenshaw and Carla Akotirene, exposes the oppressions related to race, social class, gender, and nationality, showing that colonial violence, such as racism, xenophobia, and gender inequality, influences the living conditions of migrants and refugees. Hora ^39^ shows that sexual, obstetric, and armed violence in large urban centers, in addition to institutional racism, can impact the mental health of Congolese women.

Considering the above, we sought to organize the types of violence by their nature and expressions to form a framework that can visualize the social situation of migrant women. Notably, however, the intersection of the types of violence described in each article clearly illustrates the complex submission to xenophobic, misogynistic, racist, and classist power structures, making this framework more akin to a kaleidoscopic myriad. Thus, we note that, despite the movement toward sintering studies, this perspective of violence’s intricate complexity must be considered.

Hunger, as an expression of structural violence, is a fact for millions of women and families. The sample identified migrants as experiencing violence intrinsically linked to the production of vulnerability ^19^ ^,^ ^20^ ^,^ ^36^: “*It’s been two days since I’ve eaten, both because of the pain and the lack of money. Sometimes I think that if I had a gun, I would have ended my misery; I can’t bear suffering like this anymore*” ^19^ (p. 4). This excerpt also indicates the interrelationship between hunger and fragile mental health, leading to a severe situation in which the woman interviewed in the study reported suicidal ideation.

Another issue raised, albeit rarely, concerns the direct relation between violence and the health of migrants. Arruda-Barbosa et al. ^19^ argue that sexual exploitation against Venezuelan women is linked to psychological distress, depression, and anxiety. Migrants who have experienced or are currently experiencing domestic violence report fearing their partners and, when housed in shelters, also fearing other men with whom they share the space, especially when they need to use the restrooms there ^22^. Domestic violence is also related to depression, stomach pain, shame, fear, stress, and high blood pressure ^25^. Similarly, depression is interrelated with situations of intimate partner violence ^32^. 

Underreported cases of violence and femicide, as well as errors in data entry, such as in the ethnicity/skin color field, are also a severe problem, especially in smaller municipalities, border municipalities, and municipalities with Indigenous populations ^16^. In addition, there is underreported violence against migrant women, especially those in situations of sexual trafficking and exploitation and in irregular situations, given that they are often under the control of international criminal networks ^34^. 

Human trafficking for contemporary slave labor (CSL), sexual exploitation, and forced prostitution appear in Brazilian border regions ^16^ ^,^ ^35^, with Bolivian women in CSL ^37^, with the poorest, unemployed, Black, and young women being the most exposed to this violence ^35^. In situations of sexual exploitation, being a refugee can increase racism, xenophobia, misogyny, and poverty ^19^.

Several types of gender-based violence were reported in the sample, such as sexual violence ^16^ ^,^ ^20^ ^,^ ^21^ ^,^ ^24^ ^,^ ^29^ ^,^ ^39^ and, rape ^19^ ^,^ ^24^, experienced in the workplace ^35^ and in the context of armed violence in the country of origin ^38^; psychological violence ^20^ ^,^ ^22^ ^,^ ^35^; obstetric violence ^26^ ^,^ ^37^ ^,^ ^39^; child marriage in the country of origin as a form of submission to patriarchy and gender inequalities ^17^; in domestic relationships ^18^ ^,^ ^22^ ^,^ ^29^ ^,^ ^38^, with physical and verbal abuse and psychological threats ^22^; threats with the use of sharp weapons ^19^; and physical violence ^24^ ^,^ ^35^ in domestic relationships, as noted above and overlapping with sex work ^19^. Gender inequalities among female university students were also the subject of a study ^21^.

In addition to the types of violence described above, we found interpersonal racism against Black or Indigenous women ^21^ ^,^ ^29^ ^,^ ^36^ ^,^ ^37^ ^,^ ^39^, institutional racism within health care services ^38^, and racism against Cuban health care professionals ^23^. In the latter case, racism is expressed when Brazilian women on social media question their profession - are they really doctors? - and they are identified as domestic workers ^23^. Although not mentioned in the article, we should note the misogyny intersecting with such violence.

The relations between racism, xenophobia, and mental health are addressed in three documents ^28^ ^,^ ^38^ ^,^ ^39^. In summary, we have that: “*Regarding the mental health of refugees, mental distress is not restricted to merely psychopathological or biomedical factors; it occurs due to social causes such as precariousness, injustices, losses, family separation, xenophobia, racism, and violence*” ^39^ (p. 194).

That said, we note the intersection of several forms of violence against migrant women in some studies, jeopardizing women’s mental health. Specifically, xenophobia is indicated as a relevant theme in the sample ^17^ ^,^ ^19^ ^,^ ^20^ ^,^ ^22^ ^,^ ^28^ ^,^ ^29^ ^,^ ^35^ ^,^ ^36^ ^,^ ^37^, being experienced directly by migrant women and, in some cases, by their children ^37^ ^,^ ^38^.

Political persecution in the country of origin is addressed in the literature ^36^ ^,^ ^38^ as one of the possible motivations for emigrating, in addition to structural violence ^18^ ^,^ ^21^, which forces them to move to other countries in pursuit of better living conditions ^20^. Violence is also a motivation for moving, as one woman left her home after suffering physical and moral violence in childhood due to her homosexuality ^27^.

Patrimonial violence in the workplace ^19^ ^,^ ^37^, in refugee shelters ^22^, and by intimate partners ^25^ also affects women, as do deportation threats ^37^. The Brazilian State neglects forced migration due to environmental issues, which creates precariousness in the lives of women who face deterritorialization, debt, and drug trafficking ^18^, in addition to armed violence in large urban centers, such as Rio de Janeiro ^39^. Notably, only one more recent study also shows discriminations expressed through ableism, ageism against elderly migrants, religious intolerance, racism, sexism, and heteronormativity ^20^.

The articles that show the results of studies on Brazilian women emigrants abroad contextualize and historicize coloniality and the representations of Latin American women, especially in five articles ^11^ ^,^ ^12^ ^,^ ^30^ ^,^ ^31^ ^,^ ^33^, highlighting the importance of this context and the understanding of the perverse and violent discourses surrounding the bodies of Brazilian women: “*The problem lies in the very existence of the prostitute stigma, which, related to coloniality, sexism, and racism, creates roles and imaginaries for women, with Brazilian women being considered ‘sinners, Eves, prostitutes, available, inferior, hypersexualized’. The problem, therefore, lies in the stigma of the ‘colonial body’, the available body, which affects all Brazilian women*” ^31^ (p. 880).

Brazilian women abroad also suffer gender-based violence, such as obstetric violence, with several invasive and non-consensual procedures ^11^. In this context, there are also reports of racism and xenophobia in daily life ^11^ ^,^ ^30^ ^,^ ^31^ ^,^ ^32^ and during pregnancy and childbirth. The expressions of gender-based violence experienced by Brazilian women include fetishization, hypersexualization, and objectification, as discussed above ^12^ ^,^ ^30^ ^,^ ^31^ ^,^ ^33^. Sexual violence is also noted and associated to structural issues, such as machismo and xenophobia, which increase fears and insecurities ^30^.

Despite oppression and violence, studies also note migrants’ resistance and leadership throughout their journeys. They develop strategies to find employment and care for themselves and their families, seeking to guarantee their rights. They also act in the face of racism, making it clear to perpetrators that they are racist, in addition to recognizing the racial division of labor ^38^. In the case of Brazilian women in Portugal, they face resistance and promote their agendas through networked social movements, receiving support and participation from other migrants and Portuguese women ^31^.

## Discussion

“*You broke the world*

*into several pieces and*

*called them countries*

*claimed ownership over*

*what never belonged to them*

*and left others with nothing*

*− colonized*” 

Rupi Kaur ^41^ (p. 137, free translation).

Rupi Kaur’s powerful words ^41^ resonate in this literature review, and they are associated with the migratory journeys of migrant women in Brazil and Brazilian emigrant women abroad. They face the materialization of historical processes of invasion and colonization that have escalated the countless acts of violence against their bodies. We found that violence against migrants is circumscribed by gender coloniality, in which domination, power relations, and the mandate of masculinities order abuse, psychological violence, rape, and femicide ^6^. It is also within the colonial pattern of gender that: “(...) *Women and their offspring become vulnerable and killable as never before*” ^6^ (p. 24).

The review shows that the violence associated with migration processes represents hardships that Latin American women must face. However, it also symbolizes the creation of resistance translated into actions to confront racism, xenophobia, sexism, gender-based violence, and the sexual and racial division of labor, not always with the help of support networks and state initiatives to guarantee their rights. Forced migration − which includes refugees, internally displaced persons, asylum seekers, stateless persons, and crisis and survival migrants − is characterized by intense human rights violations and violence against women ^42^. This process is not ended upon arrival in Brazil, as a destination country. Here, they encounter diverse situations of violence that have implications for their lives, health, and work.

Although some studies focus on refugee migratory status and its relation with violence and health ^19^ ^,^ ^20^ ^,^ ^29^ ^,^ ^36^ ^,^ ^38^ ^,^ ^39^, we found that forced migrations appear and are interrelated with violence, especially when they are motivated to emigrate because they are victims of violence. 

However, what is striking is the fact that, often, regardless of migratory status (being a refugee or not), violence is recurrent even when they come to Brazil to study ^21^, affirming that a single analytical category is not sufficient to understand the expressions of violence. Being intersectional and complex, it is not possible to standardize them, as we consider historical perspectives, the intersections of oppressions, whether related to nationality, social class, race/ethnicity, and migratory status, among others ^3^ ^,^ ^7^ ^,^ ^9^. 

Although intersectionality is neither shown nor conceptualized in several studies, part of the sample ^11^ ^,^ ^12^ ^,^ ^19^ ^,^ ^24^ ^,^ ^25^ ^,^ ^29^ ^,^ ^31^ ^,^ ^32^ ^,^ ^33^ ^,^ ^36^ ^,^ ^37^ ^,^ ^38^ ^,^ ^39^ shows compositions of expressions of violence and their relations with structural inequities, whether in the country of origin or Brazil. Thus, as an analytical, theoretical, and methodological tool ^7^, intersectionality is crucial for studies on migrant women, including for proposing effective public policies that understand them as protagonists of their very displacements ^9^. 

Studies show an intersection between living conditions and productive work - exploitation and precarious, often unhealthy and poorly paid activities - and reproductive conditions, with domestic and care work overload, and the mental health of migrant women, who experience accumulated distress, health symptoms, and illnesses. Different forms of violence intersect these processes, further compromising their lives. We found some extreme cases with women desiring to end their lives as a way to end the distress ^19^, even when femicide does not reach them first ^16^.

Regarding the work of these migrants, the review indicates that several experiences resemble or are expressions of conditions analogous to contemporary slavery and sexual exploitation, influencing their physical or mental illnesses in the face of labor exploitation and workplace violence. As women socially responsible for care work, they tend to care for their children and families without support networks or the Brazilian State, raising their children, for example, in workshops. This setting and family life intertwine, especially in the situation of those who work in sewing workshops ^43^. 

By correlating mental distress and illness with women’s life factors, these studies build a perspective in which mental health is deindividualized. That is, rather than being understood as a solely and exclusively personal attribute, it is considered socially determined by life conditions. In other words, living conditions, work, housing, food, movement through spaces, protection, documentation, access to services, and exposure to violence, such as racism and xenophobia, constitute a framework of social determination of the health-disease process that degrades and weakens the mental and physical health of migrant women.

If we consider the subjective and material place through which the women in this sample circulate, who are mostly Latin American and seek protection and better living conditions for themselves and their families, we must add the weight of the colonial legacy that subjugates them in a matrix of power that fosters and reproduces social inequality and the spaces they are allowed to occupy, especially when they accumulate marks of subjection ^44^. In other words, race, gender, sexuality, social class, and place of origin hierarchize and increase vulnerabilities the further these women move away from the hegemonic individual form, notably in societies with racist, sexist, patriarchal, and xenophobic foundations.

According to Lélia Gonzalez ^45^, these are indelible marks of the construction of Brazil, which encompass all spheres of individual and collective life, that is, they have direct and indirect effects on individuals and communities, embedding oppressions that foster stereotypes that naturalize sexual exploitation, unhealthy and poorly paid work and the overload of reproductive work for some groups of women, notably Black women.

As we consider that social relations and colonialist capitalist forms influence the living conditions of migrants ^9^ ^,^ ^46^, it is not possible to homogenize their experiences or disregard the contradictions between the advances and setbacks in their displacements. As for mental health, in addition to the experiences of migratory processes, there is a set of situations, such as economic ones, lack of documentation, lack of social and family support, etc., that can be related to the demands for mental health care. This fact is not always understood by health care services and professionals, who may associate these plural and singular histories with a pathologizing perspective ^46^.

The contradictions in migrants’ experiences with Brazilian health care services are striking. While some studies praise the SUS as a valuable path to the right to health, countless barriers are identified. Access to and retention in health care networks are influenced by linguistic barriers, institutional violence, racism, and xenophobia experienced within services.

Thus, health care professionals require training to care for migrants, which is one of the recommendations for health care policies in the SUS. A recent technical note from the Brazilian Ministry of Health on the care of migrants in primary health care states that, to guarantee health care for this population, it is necessary to respect their culture and language, provide care even when they lack personal documents, and provide documents in multiple languages, among other principles ^47^.

Culturally sensitive and truly caring health care practices need to recognize and adopt the theoretical and methodological perspective of intersectionality ^7^ in order to understand health and illness processes more broadly. In mental health, this perspective is essential to escape a reductionist determinism that holds individuals responsible for their health, necessarily implying socially (re)produced macro-, meso-, and micropolitical factors. It also provides important support for public initiative, based on the recognition and assumption of responsibility for training attentive, sensitive, receptive, and more effective services and professionals regarding the care and consideration of migrant women, tackling the violence that affects them and protecting their physical and mental health.

Given the complexity of the social and historical relationships involving migrants, the proposal for intercultural care in the health field values ​​reflections on the sociocultural practices and processes existing among people and groups, understanding their heterogeneities and the influences of social inequalities, racism, and structural violence on health care. It is fundamental that public health policies ensure greater popular participation for their effective implementation ^46^ ^,^ ^48^. 

Based on this literature review, we suggest further theoretical and methodological exploration of the agency of migrants, who, day after day, in their social movements, seek the right to health and work, free from violence and certain that they are the protagonists of their own stories, as affirmed by research on intersectionality and Black and/or decolonial feminisms ^6^ ^,^ ^7^ ^,^ ^9^ ^,^ ^45^. 

Another approach presented in the review concerns Brazilian women abroad. As feminists, we are struck by the fact that most articles refer to the fetishization and hypersexualization of Brazilian women in the Global North. Being far from home, in a different culture, living in a territory where the coloniality of gender was born ^6^, makes us reflect on the distress and challenges women face in their daily struggle for better living conditions for themselves and their families. Furthermore, this context raises questions about public health policies geared toward them: What are these policies? Who provides health care services, given their role as migrants? On the other hand, we understand that their resistance movements echo throughout the world: “*Their voices speak of experiences of being expatriate women, intertwined with macrosocial structures, and connected to past struggles, revisited from a different feminist perspective, one that has turned the world upside down*” ^33^ (p. 23).

Despite the heterogeneous contexts of Brazil and the countries of the Global North to which Brazilian women migrate, we believe that one of the differences lies in the fact that our compatriots do not always have access to public health care services, as is the case with SUS, which, despite its challenges, serves part of the migrant population in the country. Among the similarities, violence undoubtedly stands out, highlighting the need for care throughout their migrations.

One limitation of this study is its lack of quality assessment of the research presented here. The limited literature on Brazilian emigrant women abroad also limited its reach beyond Portugal and England. However, this path could be explored in future research, expanding consideration to the violence that affects Brazilian women, especially when seeking new opportunities in the Global North. 

## Final considerations 

The research presented highlights intersectional violence against migrant women in Brazil and Brazilian emigrant women abroad, understanding that generalizations are impossible because their lives and stories are diverse. However, it shows that violence is related to gender, race, social class, nationality, generation, disability, and migratory status, as indicated by intersectional studies that rightly note the intricate interplay of oppressions that make them vulnerable.

Regarding health and illness processes, we found that living and working conditions, food security, housing, mobility and access (or lack thereof) to health care and social care services, and situations of violence (racism, xenophobia, gender-based violence, ageism, political persecution, ableism, among others) are interrelated with physical and mental illnesses, which often impact the personal and familial well-being and care.

The dynamics of household work, which has historically been the responsibility of women, especially Black and Indigenous women, was one focus presented in the review. Therefore, unpaid or underpaid work performed by women should be taken into account when considering the health of migrant women, as work overload is a fact. Despite the challenges posed by subjection to oppression and violence systems, the review shows resistance and protagonism among migrants throughout their journeys. These strategies are designed to guarantee their rights as migrants and women. In this regard, they are not alone. Through collective movements, they confront and embrace resistance to colonial violence in a world where they “*claim ownership over what never belonged to them and leave others with nothing*” as stated by Rupi Kaur ^41^ (p. 137, free translation).

## Data Availability

The databases used in the study, including extraction codes, analyses, and results, are available in the repository: (https://osf.io/c9vba/overview).
