# Supplementary material for: Intersectional violence and the health of migrant women in Brazil and Brazilian emigrant women abroad: a scoping review
Source: Cad Saude Publica. 2026 May 18;42:e00197625. doi: 10.1590/0102-311XEN197625 (PMC13207380; doi:10.1590/0102-311XEN197625)
Supplement: Translation

# Translation: Violências interseccionais e a saúde de mulheres migrantes no Brasil e de brasileiras no exterior: uma revisão de escopo

**DOI:** 10.1590/0102-311XPT197625

**Authors:** Cristiane Batista Andrade, Fernanda Mendes Lages Ribeiro, Corina Helena Figueira Mendes, Rosana Evangelista-Poderoso, Bárbara Santos Leitão, Maria Isabel Ramos da Silva Abelson

**Affiliations:** 1 Escola Nacional de Saúde Pública Sergio Arouca, Fundação Oswaldo Cruz, Rio de Janeiro, Brasil.; 2 Instituto Nacional de Saúde da Mulher, da Criança e do Adolescente, Fundação Oswaldo Cruz, Rio de Janeiro, Brasil.; 3 Faculdade de Ciências Médicas, Universidade Estadual de Campinas, Campinas, Brasil.

**Keywords:** Migração Humana, Mulheres, Enquadramento Interseccional, Violência, Saúde de Migrantes

## Abstract

Os fluxos migratórios têm se intensificado com o aumento da migração de mulheres da América Latina e de outros continentes para o Brasil, bem como com a emigração de brasileiras para o Norte Global. Considerando a realidade da feminização das migrações e a importância de compreender as opressões vivenciadas pelas mulheres em face aos seus deslocamentos, esta revisão de escopo analisa, sob uma perspectiva interseccional, as evidências científicas sobre a saúde e as expressões de violências contra mulheres migrantes no Brasil e mulheres brasileiras no exterior. As bases de dados utilizadas foram BVS, Cochrane, Embase, PubMed, SciELO, Scopus e Web of Science, empregando-se descritores relacionados à temática. Ao todo, 26 documentos foram incluídos para análise. Sobre a saúde das migrantes, os resultados indicam que a migração tem implicações para o acesso e a atenção à saúde, a saúde sexual e reprodutiva, o adoecimento mental e a manifestação de sofrimento, além das barreiras de acesso aos serviços de saúde decorrentes de aspectos interculturais e linguísticos. Tanto as migrantes no Brasil quanto as brasileiras no exterior sofrem com as violências interseccionais, incluindo expressões de racismo, xenofobia, desigualdade racial e de gênero no mercado de trabalho, violência obstétrica, sexual e patrimonial, e condições análogas à escravidão, somadas à hipersexualização das mulheres brasileiras no Norte Global. Contudo, apesar dos desafios, opressões e violências, a revisão revela as resistências e os protagonismos das migrantes ao longo de suas trajetórias, à medida que estratégias individuais e coletivas são desenvolvidas para garantir seus direitos enquanto migrantes e mulheres.

## Introdução 

Os fluxos migratórios têm se intensificado na América Latina, especialmente no Brasil. Dados oficiais brasileiros indicam que 481 mil pedidos de residência foram regularizados no país de 2022 a julho de 2024 e que, embora os homens sejam a maioria, o número de mulheres, crianças e adolescentes cresceu significativamente, sendo as nacionalidades mais prevalentes a venezuelana, a boliviana, a colombiana e a argentina ^1^. Nesse mesmo período, aproximadamente 139.200 pessoas solicitaram refúgio no país, com predominância de venezuelanos, mas também incluindo cubanos e angolanos: “...*96,0% delas de nacionalidade venezuelana, com as mulheres representando 47,2% dos refugiados*” ^1^.

É fundamental elaborar estudos e produzir dados (empíricos, teóricos e metodológicos) sobre a migração de mulheres, sobretudo no que diz respeito às suas motivações, uma vez que, historicamente, elas têm sido vistas como coadjuvantes de seus companheiros, e não como protagonistas de sua própria história ^2^. 

É cada vez mais comum que as mulheres migrem sozinhas ou acompanhadas de seus filhos, tendendo a buscar emprego para sustentar suas famílias ^2^. Portanto, no Brasil, enquanto país que tem recebido uma grande diversidade de migrantes, especialmente nas últimas décadas, deve-se compreender a violência interseccional ^3^ contra mulheres migrantes com o intuito de prover cuidados de saúde, prevenir situações de violência e desenvolver políticas públicas eficazes para o enfrentamento desse grave problema de saúde pública.

O protagonismo e os desafios que as mulheres enfrentam no deslocamento humano vêm sendo cada vez mais discutidos sob uma perspectiva feminista, elucidando o movimento crescente e as diversas razões para a migração ^4^. Embora a partida de sua terra natal seja motivada por uma privação de direitos, as mulheres deparam-se com políticas de mobilidade social limitadas para a melhoria de suas condições de vida, o que contribui para o aumento de suas vulnerabilizações ^5^.

Além disso, nos contextos migratórios, as violências podem ser exacerbadas, evidenciando como a migração se entrelaça com o colonialismo e a subalternizações dos corpos ^4^. Historicamente, as violências contra as mulheres estão ligadas à dominação masculina e foi agravada pela colonização, na qual a racialização e a sexualização das relações de gênero caracterizam as estruturas forjadas desde a invasão europeia na América Latina. Dentro do padrão colonial de gênero, a crueldade, a impunidade e a naturalização das violências contra as mulheres são cada vez mais prevalentes, afetando suas vidas, incluindo os casos de feminicídio ^6^.

Nesse sentido, os estudos interseccionais fundamentam as análises das opressões e violências contra as mulheres, como o sexismo, o racismo e o classismo, que tornam complexas as dinâmicas das violências. Por essa razão, aborda-se as diversidades entre as mulheres, inclusive entre aquelas de uma mesma classe social e etnia. Em outras palavras, analisar como as intersecções das opressões moldam as suas experiências é relevante para o desenvolvimento de políticas públicas voltadas a atender às suas necessidades e a enfrentar as violações de direitos e as violências ^5^ ^,^ ^7^. 

Com relação aos processos migratórios, há um consenso de que eles impactam a vida das mulheres de maneira diferenciada, a depender de sua nacionalidade, *status* migratório, orientação sexual, identidade de gênero, raça/etnia e classe social ^5^ ^,^ ^8^ ^,^ ^9^. Consequentemente, ao utilizar a interseccionalidade como eixo de análise, é possível explorar com maior profundidade as experiências das mulheres migrantes, compreendendo suas opressões e resistências para além das relações assimétricas de gênero, incluindo também as distinções e discriminações baseadas em raça e classe social, o acesso ao emprego e as condições de migração, a afinidade com o idioma do país de destino, entre outros indicadores ^8^ ^,^ ^9^.

Assim, considerando que as violências são interseccionais e complexas, Collins ^3^ reitera que se trata de um problema social que acarreta sofrimento e danos àquelas que a vivenciam. A autora acredita que, para além das intersecções das violências de raça, gênero, nacionalidade e classe social, é crucial identificar os seus determinantes no contexto da exploração em uma sociedade capitalista, especialmente em países que sofreram com a colonização ^3^, característica da região latino-americana. Portanto, ao considerar essas reflexões no contexto das mulheres migrantes, infere-se que as mais empobrecidas, negras, indígenas e oriundas do Sul Global são as mais afetadas pela violência racial e de gênero, perspectiva esta adotada no presente artigo.

Se os deslocamentos de mulheres envolvem inúmeros desafios, como se interrelacionam os processos migratórios e sua saúde? Uma revisão recente da literatura ^10^ constatou que, além das dificuldades migratórias, as mulheres também enfrentam vulnerabilizações e desigualdades sociais e econômicas exacerbadas, vínculos empregatícios precários e a falha dos serviços em atender às suas necessidades de saúde, especialmente durante a pandemia. As mulheres encontram barreiras para conciliar o trabalho produtivo com o cuidado familiar, o que se expressa pela sobrecarga de demandas, impactando sua saúde física e mental. Elas são frequentemente submetidas às violências racial, de gênero e xenofóbica, o que pode causar ou agravar o sofrimento e os transtornos psicológicos ^10^. 

Contudo, essa violência não se restringe ao Brasil. Elas também são vivenciada por mulheres brasileiras no exterior que, ao decidirem migrar para o Norte Global, relatam sofrer racismo e xenofobia, além da hipersexualização de seus corpos, estigmatizados como “fáceis” e sensuais ^11^ ^,^ ^12^, somadas a expressões de violência obstétrica ^11^. Dessa forma, elucidar como nossas conterrâneas experienciam os processos migratórios e o acesso aos cuidados de saúde no Norte Global é importante para revelar as violências ^11^ e o padrão colonial de gênero que se perpetua ^6^. Essa perspectiva reforça a Lei de Migração (*Lei nº 13.445/2017*) ^13^, que incentiva a produção de estudos e pesquisas sobre mulheres brasileiras no exterior, visto que essa população é uma responsabilidade do Estado brasileiro.

Assim, esta revisão de escopo tem como objetivo analisar as evidências científicas sobre a saúde e as expressões das violências contra mulheres migrantes no Brasil e mulheres brasileiras no exterior. A questão de pesquisa é: Quais são as evidências científicas sobre a saúde e as expressões das violências contra mulheres migrantes no Brasil e mulheres brasileiras no exterior?

## Método

Esta revisão de escopo tem como objetivo apresentar uma visão ampliada sobre o tema, discutir a diversidade de conhecimentos produzidos, identificar lacunas e subsidiar futuras revisões sistemáticas ^13^. Foram seguidas as recomendações do *Reporting Items for Systematic Reviews and Meta-Analyses: Extension for Scoping Reviews* (PRISMA-ScR; Itens de Relatórios Preferenciais para Revisões Sistemáticas e Metanálises: Extensão para Revisões de Escopo) ^14^ ^,^ ^15^, que preveem o registro prévio do protocolo da revisão de escopo, realizado em outubro de 2024 (https://osf.io/c9vba/overview), no qual podem ser consultadas as bases de dados e os descritores utilizados. A estratégia de busca foi elaborada por bibliotecária com doutorado em Ciências da Saúde e experiência nas principais bases de dados da área da saúde e interdisciplinares.

Após a definição do objetivo e da questão de pesquisa, foram estabelecidos os critérios de população, conceito e contexto (PCC) da revisão ^14^: (a) população: mulheres migrantes (jovens maiores de 18 anos, adultas e idosas); (b) conceito: violências e cuidado em saúde; e (c) contexto: o Brasil como país de destino de migrantes transnacionais e outros países de migração de mulheres brasileiras. 

O Quadro 1 apresenta as bases de dados consultadas - SciELO, Biblioteca Virtual em Saúde (BVS), Embase, Web of Science, Scopus, Cochrane e PubMed -, os campos de busca utilizados e o número de documentos identificados.

**Quadro 1:** Bases de dados consultadas, campos utilizados e número de documentos.

BASES DE DADOS ADOTADAS	CAMPOS UTILIZADOS	DOCUMENTOS
BVS	Título, assunto e resumo	137
Cochrane	Título, assunto, resumo e descritor MeSH	4
Embase	Todos os campos, EMTREE	67
PubMed	Título/Resumos, termos MeSH, todos os campos	443
SciELO	Todos os índices	71
Scopus	Título, assunto e resumo	45
Web of Science	TS = tópico e AB = resumo	269
Total com as duplicatas		1.036
Total após exclusão automática de duplicatas via Zotero		899
Total após exclusão manual de duplicatas na plataforma Rayyan		895
Total sem duplicatas		895

Fonte: elaborado pelas autoras.

Os documentos recuperados nas bases (n = 1.036) foram inseridos no software de gerenciamento de referências Zotero (https://www.zotero.org/) para identificação e remoção de duplicatas, totalizando n = 899 após essa etapa. Em seguida, esse conjunto foi importado para o sistema Rayyan (https://www.rayyan.ai) e a ferramenta de detecção de duplicatas foi novamente acionada, identificando mais quatro registros duplicados e resultando em uma amostra final de n = 895. Nesse mesmo sistema, realizou-se o processo de seleção em duplo mascaramento por duas pesquisadoras independentes, sem necessidade de terceira avaliadora, uma vez que os eventuais conflitos foram resolvidos por consenso.

Foram incluídos artigos, livros, teses e dissertações que: (a) abordassem saúde e violência contra mulheres migrantes (jovens maiores de 18 anos, adultas e idosas) no Brasil ou mulheres brasileiras no exterior; (b) estivessem disponíveis online e na íntegra; e (c) estivessem redigidos em português, inglês ou espanhol. Foram excluídos trabalhos que: (a) tratassem de temáticas alheias ao escopo da pesquisa; (b) abordassem homens, mulheres, crianças ou adolescentes; e (c) não estivessem disponíveis na íntegra. A Figura 1 apresenta o fluxograma da revisão conforme o PRISMA ^15^.

**Figura 1:** Fluxograma do PRISMA para a seleção de documentos para a revisão de escopo.

## Resultados 

Após a definição dos estudos incluídos para análise, foi elaborado o Quadro 2, contendo os principais resultados das pesquisas acerca das expressões das violências e da saúde de mulheres migrantes.

**Quadro 2:** Síntese dos artigos selecionados: nacionalidade das migrantes, localização no Brasil e no exterior, objetivo, metodologia, principais resultados sobre saúde e expressões de violência.

ESTUDO (ANO)	POPULAÇÃO, CONTEXTO E PERFIL DAS PARTICIPANTES E LOCALIZAÇÃO NO BRASIL (PCC)	OBJETIVO	MÉTODOS	CONCEITO PRINCIPAIS RESULTADOS EM SAÚDE	CONCEITO PRINCIPAIS RESULTADOS SOBRE EXPRESSÕES DE VIOLÊNCIA
MIGRANTES TRANSNACIONAIS NO BRASIL					
1. Meneghel et al. ^16^ (2022)	Mulheres de diferentes nacionalidades nas fronteiras brasileiras	Analisar os óbitos de mulheres por agressão nos 122 municípios da faixa de fronteira brasileira, mensurar e identificar fatores associados	Quantitativa − dados do SIM. Variáveis independentes: migração, concentração de renda, raça/cor da pele, população, religião e violência sexual	O estudo aprofundou pouco a discussão sobre saúde. Contudo, mostra que as mulheres indígenas, além de sofrerem com a violência (13% de 181 feminicídios), enfrentam graves problemas de saúde. Reitera que os processos migratórios influenciam a saúde das populações	As regiões de fronteira podem ser afetadas pelo tráfico de drogas, armas e pessoas, levando ao trabalho escravo contemporâneo, exploração sexual, prostituição forçada, feminicídio e homicídio. Foram registrados 1.384 óbitos femininos de 2000 a 2015, com taxa média de 5,8/100.000. O padrão de distribuição desses óbitos concentrou-se predominantemente no Arco Central (Rondônia, Mato Grosso e Mato Grosso do Sul), com os maiores contingentes de migrantes e relatos de violência sexual, especialmente em municípios maiores e mais populosos. Houve um aumento em municípios de pequeno porte. Há subnotificação de casos, especialmente entre mulheres indígenas
2. Ministério da Saúde ^34^ (2013)	Profissionais de saúde, assistentes sociais e de outros setores envolvidos com migração e violência contra as mulheres	Compor o material didático para o curso “Migração e Tráfico de Mulheres para Exploração Sexual e Trabalho Degradante” para promover a capacitação de profissionais da saúde, assistência social e de outros setores governamentais e não governamentais	Qualitativa − dados da literatura, do SIM e de outras bases de dados em saúde	A saúde das mulheres migrantes é afetada por condições insalubres, falta de acesso aos serviços de saúde e exposição a riscos e doenças ocupacionais. Destaca-se a responsabilidade dos serviços de saúde na identificação e intervenção em situações de violência contra as mulheres	O tráfico de pessoas afeta mulheres pobres, desempregadas e jovens, que são as mais vulneráveis ao trabalho forçado e à exploração econômica ou sexual. Estão mais expostas à migração entre estados brasileiros ou para outros países, tornando-se vulneráveis em situações de violência, exploração sexual comercial no caso de adolescentes ou crianças, e prostituição forçada quando adultas, com ou sem pagamento mínimo por seu trabalho
3. Ministério da Saúde ^35^ (2013)	Profissionais de saúde, assistentes sociais e de outros setores envolvidos com a migração e violência contra mulheres	Compor o material didático para o curso “Migração e Tráfico de Mulheres para Exploração Sexual e Trabalho Degradante” para promover a formação de profissionais da saúde, assistência social e de outros setores governamentais e não governamentais. O livro contém 11 artigos sobre o tema e aborda brasileiras no exterior	Qualitativa − dados da literatura e da experiência do projeto Suindara, para capacitação profissional no SUS	Os impactos da violências na saúde incluem exploração sexual, trabalho degradante, dificuldades na atenção à saúde sexual e reprodutiva, sofrimento psicológico e transtornos mentais, dependência química, condições precárias de vida e saúde, maus-tratos, tráfico de pessoas em áreas rurais e urbanas, vulnerabilidade material e transferência de pessoas para lugares sem vínculos afetivos, comunitários e sociais. A ruptura das relações sociais, afetivas e simbólicas, as dificuldades de integração cultural, o isolamento, a solidão e as pressões e tensões do cotidiano, subjacentes ao processo migratório e à situação de irregularidade, podem levar a estados de fragilidade psicológica, ocasionando sofrimento psicológico e transtornos mentais	As mulheres migrantes estão entre as mais vulnerábilizadas à violência sexual no local de trabalho, e as mulheres negras são as principais vítimas do tráfico de pessoas, especialmente na região de fronteira brasileira. As mulheres vivenciam violência institucional nos setores de saúde e assistência social e sofrem violência de gênero, sexual, física, psicológica, coletiva e xenofóbica
4. Oliveira ^36^ (2022)	Mulheres bolivianas, haitianas, angolanas, congolesas e venezuelanas em São Paulo	Discutir os processos migratórios e a saúde mental de mulheres migrantes e refugiadas em São Paulo	Qualitativa − questionário e entrevistas com nove migrantes	O sofrimento psicossocial, as situações de vulnerabilizadas e as opressões baseadas em gênero, raça e classe social apontam para os efeitos adversos na saúde mental das migrantes. As mulheres têm rotinas exaustivas, com poucos momentos de lazer. Os afazeres domésticos e o cuidado familiar estão associados a essa sobrecarga. Enfrentam discriminação nos serviços de saúde por serem migrantes; há sofrimento relacionado aos rompimentos familiares, com tristeza, medo e preocupações com a subsistência familiar deteriorada pela Covid-19. São relatadas crises de ansiedade, distúrbios do sono, cefaleias, ataques de pânico e uso de psicotrópicos	Racismo, discriminação, inclusive nos serviços de saúde, xenofobia, assédio moral, fome e perseguição política no país de origem e abusos verbais afetam as mulheres
5. Avellaneda Yajahuanca ^37^ (2015)	Mulheres bolivianas em São Paulo	Analisar as experiências de mulheres bolivianas durante a atenção à saúde, parto e pós-parto	Qualitativa − observação participante e entrevistas individuais	A assistência pré-natal e pós-parto é precária, aliada a graves barreiras linguísticas e culturais. Devido ao trabalho em oficinas, dormem mal, o ambiente de trabalho é o mesmo da casa, a alimentação é ruim, há pouca ventilação e a poeira dos tecidos afeta a saúde respiratória. Distúrbios musculoesqueléticos estão relacionados à costura e à fadiga. As oficinas são precárias, com problemas elétricos, vazamentos de água e mofo. Em relação ao parto, sentem-se mais seguras em casa. Há fragilidades no cuidado, que desconsideram dimensões culturais durante a gravidez e o puerpério	Foram relatadas violência obstétrica (manobra de Kristeller, episiotomia, uso de fórceps; repetidos exames vaginais; dificuldades de interação com profissionais de saúde devido a barreiras culturais e linguísticas; violência institucional com expressões xenofóbicas por profissionais; racismo); violência doméstica; falta de licença-maternidade; violência financeira (roubos) nas oficinas; jornadas de trabalho exaustivas, falta de pagamento, casos de trabalho análogo à escravidão contemporânea e falta de direitos trabalhistas. Há ameaças de deportação por parte de seus empregadores. Seus filhos sofrem bullying e/ou xenofobia
6. Balestro & Pereira ^17^ (2019)	Mulher gambiana no Rio de Janeiro	Discutir língua e cultura no contexto da migração feminizada	Qualitativa − recursos audiovisuais disponíveis online (entrevistas) com uma refugiada, além do uso de dados oficiais sobre o *status* de refúgio	A entrevistada descreve sua motivação para deixar seu país como resultado de seu desejo de estudar, ter uma profissão e ser independente. Há dificuldades com o idioma no Brasil e a necessidade de quebrar barreiras no acesso à educação, saúde e outros serviços públicos, especialmente nos pontos de entrada de migrantes	A entrevistada aborda a cultura do casamento infantil em seu país, bem como as relações patriarcais que reverberam nas desigualdades de gênero, além da migração forçada e xenofobia
7. Calderón Uribe et al. ^18^ (2021)	Mulheres brasileiras no contexto de deslocamento interno (Porto Alegre, Rio Grande do Sul)	Compreender as perspectivas de mulheres brasileiras deslocadas internamente, que são chefes de família, e as ações do Estado face a essa migração	Qualitativa − estudo de caso com análise de discurso de três entrevistas com mulheres que vivenciaram o deslocamento em Porto Alegre	O deslocamento forçado, as condições precárias de moradia, a violência estrutural e a falta de políticas públicas efetivas que atendam às necessidades de saúde das mulheres têm implicações diretas na sua saúde física e mental e na de suas famílias. A falta de um sistema de saúde integrado e acessível a essa população é uma das principais deficiências observadas no âmbito da assistência social em resposta ao deslocamento. Identificou-se um caso de mulher em situação de rua	As mulheres entrevistadas e suas famílias enfrentaram diversas formas de violência durante seu deslocamento, além da migração forçada devido a questões ambientais. Essa violência pode se manifestar de forma física, direta, indireta e simbólica. Suas experiências e narrativas são desvalorizadas e tornam-se uma forma de violência perpetuada pela negligência e falta de reconhecimento por parte do Estado. A insegurança persistente, a urbanização excludente e as condições precárias de moradia são vistas como consequências da violência estrutural que perpetua a pobreza e exacerba a vulnerabilidade a novos deslocamentos. Há relatos de violência doméstica e morte de filhos e companheiros em decorrência do narcotráfico
8. Arruda-Barbosa et al. ^19^ (2024)	Mulheres venezuelanas no Estado de Roraima	Analisar as violências perpetrada contra mulheres venezuelanas imigrantes profissionais do sexo a partir de uma análise interseccional	Qualitativa − entrevistas com profissionais do sexo venezuelanas e reportagens da mídia online sobre o tema	As condições precárias de saúde decorrem da vulnerabilidade social e econômica. A violência e a exploração sexual também causam sofrimento psicológico e adoecimento mental, desencadeando depressão e ansiedade. Oferecer pagamento adicional por sexo sem uso de preservativo aumenta a exposição a Infecções Sexualmente Transmissíveis (ISTs) e outros problemas de saúde. A saúde da mulher reflete diretamente a condição de migração associada às desigualdades de gênero e raciais, evidenciando a necessidade de atenção à saúde e as interfaces com essas opressões	Violência estrutural em que o desemprego, a pobreza, a fome e a necessidade de subsistência são uma realidade para as mulheres venezuelanas, sendo a violência física a mais temida, pois são agredidas, inclusive com o uso de armas brancas por clientes, parceiros e até terceiros. Há violências de gênero e sexual (estupro), violência psicológica, violência verbal e exploração sexual, todas agravadas pela migração forçada, pobreza e opressões relacionadas a raça/etnia; violência patrimonial (roubo de dinheiro) e discriminação com baixos salários pelo fato de serem venezuelanas
9. Fernandes & Onuma ^20^ (2024)	Mulheres de diferentes nacionalidades no Brasil	Identificar as violências para a implementação de políticas públicas	Qualitativa − levantamento bibliográfico, análise documental e dados oficiais do Comitê Nacional para os Refugiados (CONARE) sobre pedidos de refúgio no Brasil	Situações de estresse pós-traumático decorrentes da migração forçada e adaptação ao novo país, agravadas pela vulnerabilização socioeconômica, são desafios e impactos na saúde mental das refugiadas. As barreiras de acesso à saúde são uma realidade para muitas mulheres em situação de refúgio, refletindo a negligência dos dados estatísticos brasileiros, nos quais essas mulheres são frequentemente tratadas como um grupo homogêneo, negando a necessidade de atenção às suas especificidades e realidades plurais, bem como às diferentes intersecções possíveis de opressão	Há relatos de discriminação, migração forçada e violência sexual, cultural e psicológica; violência relacionada à maternidade, discriminação e falta de acesso aos serviços de saúde, afetando a saúde física e emocional; separação de familiares pela falta de infraestrutura nos países de destino, além de dificuldades com a maternidade (gravidez, puerpério e cuidado com os filhos/familiares); discriminação interseccional baseada em classe social, gênero (identidade de gênero e orientação sexual), raça/etnia, capacitismo, intolerância religiosa e etarismo (contra mulheres migrantes idosas), e falta de garantias de direitos básicos, acentuando as vulnerabilizações econômicas, sociais, políticas e culturais destas mulheres
10. Ferreira et al. ^21^ (2022)	Mulheres migrantes colombianas, haitianas, venezuelanas e uruguaias em Foz do Iguaçu	Compreender os impactos psicológicos da migração na vida de universitárias migrantes	Qualitativa − descritiva e exploratória. Entrevistas com roteiros semiestruturados e formulário sociodemográfico intercultural e análise de conteúdo	A saúde psicológica das mulheres que migram para estudar é diretamente impactada, desde o processo migratório até sua passagem pela universidade: sofrimento psicológico, estados depressivos e ansiosos, somatização, tristeza, baixa autoestima e ganho de peso. A maternidade transnacional também exacerba o sofrimento psicológico (medo, ansiedade, solidão). As universidades precisam se comprometer com políticas de equidade de gênero, acolhimento e permanência, baseadas em uma análise interseccional. O estudo também destaca a resiliência e a autonomia das mulheres que migram	Relatos de discriminação relacionada ao espaço masculinizado de alguns cursos universitários; situações de violências de gênero e sexual desde o processo migratório até a sua permanência dentro e fora da universidade; violência estrutural, abusos e microabusos relacionados a raça, gênero, etnia, nacionalidade, cultura, idioma, entre outros; migração forçada entre mulheres venezuelanas, exploração no trabalho com baixos salários e intensas jornadas (14 horas/dia) e moradias precárias, e racismo contra uma mulher haitiana que se descobre negra no Brasil, relatando muito sofrimento, e contra uma mulher colombiana que possui ascendência indígena
11. Makuch et al. ^22^ (2021)	Mulheres venezuelanas no Estado de Roraima	Analisar experiências de violências contra mulheres venezuelanas em abrigos no Estado de Roraima	Qualitativa com grupo focal	Face às violências sofridas, as mulheres relataram medo de seus parceiros e de serem abandonadas por eles, bem como sentimentos de culpa. Expressaram também medo de outros homens que viviam nos abrigos, particularmente em relação ao uso dos banheiros	Relatos de violência doméstica (agressão física e verbal e ameaças psicológicas), machismo e xenofobia fora dos abrigos; violência perpetrada contra crianças pelos parceiros, cometida por mulheres que agrediram seus parceiros e outras mulheres que mantinham relacionamentos afetivos com eles; e violência patrimonial, física e verbal perpetrada por venezuelanos nos abrigos. Algumas conhecem a Lei Maria da Penha e a utilizam como estratégia de autoproteção
12. Santos ^38^ (2019)	Mulheres congolesas no Rio de Janeiro	Refletir sobre o processo de refúgio e a saúde mental de mulheres congolesas	Qualitativa − observação participante e entrevistas com refugiadas congolesas	As mulheres vivenciam sofrimento social e más condições de vida, depressão, ansiedade, solidão e tristeza. Há uma estreita relação entre suas experiências como refugiadas e desemprego, dificuldades linguísticas e solidão, com casos de esgotamento emocional, sofrimento, cefaleias, insônia, estresse, taquicardia, fadiga, luto, sintomas depressivos e psicossomáticos agravados pela violência armada nas favelas onde vivem. A migração forçada e a perseguição política podem causar traumas, medo, sofrimento, separação familiar, desânimo, angústia e estresse. Há relatos de lentidão no atendimento à saúde. A fé e a espiritualidade são recursos utilizados para apoiá-las frente aos desafios que enfrentam	Os relatos incluem violência doméstica; violência armada/urbana com sentimentos de medo, angústia e choro diante de assaltos e pessoas armadas; migração forçada; confrontos armados na terra natal; perseguição política; xenofobia e divisão racial no trabalho; violência sexual em confrontos armados na terra natal; racismo, inclusive nos serviços públicos de saúde, e xenofobia sofrida pelos filhos em escolas brasileiras
13. Pinto & Amaral ^23^ (2016)	Mulheres cubanas	Analisar textos e reportagens sobre a mobilidade de médicas cubanas no Brasil	Qualitativa − análise documental em sites governamentais, mídias comerciais e não comerciais sobre migração (de 2003 a 2013)	Embora o artigo não aborde a saúde das médicas cubanas, trata indiretamente do trabalho dessas profissionais de saúde	Relatos de racismo por parte de brasileiros ao questionarem se as médicas eram realmente médicas, expressos na mídia: “Essas médicas cubanas parecem empregadas domésticas”, ou “Elas são mesmo médicas?”
14. Santos et al. ^24^ (2015)	Mulheres bolivianas em São Paulo	Identificar as características de mulheres bolivianas com gestação decorrente de estupro	Qualitativa − estudo retrospectivo, e quantitativa − estudo documental com 38 mulheres bolivianas vítimas de violência sexual em um serviço público de aborto legal	O desconhecimento dos serviços de saúde pública, os vínculos empregatícios informais e a migração irregular acabam impedindo o acesso das mulheres aos serviços de saúde. Os autores apontam para subnotificações de casos de violência sexual, bem como dificuldades na busca pelo aborto legal. A maioria das mulheres chegou ao hospital após encaminhamento da polícia e de outras unidades de saúde	Ocorrência de violência sexual (estupro; 63,2% por desconhecidos), violência física e ameaças
15. Serrano & Martin ^25^ (2022)	Mulheres bolivianas em São Paulo	Analisar a violência doméstica contra trabalhadoras bolivianas em oficinas domiciliares de costura	Qualitativa - etnografia, entrevistas, observação participante e grupo focal	As mulheres são impedidas por seus empregadores de procurar os serviços de saúde; têm pouco acesso a alimentação adequada e lhes é negada a licença-maternidade. Com relação ao acesso aos serviços, enfrentam obstáculos linguísticos e culturais, como representações do processo de saúde e cuidado que diferem daquelas da sociedade brasileira. Diante da violência doméstica, relataram sofrer de depressão, dores de estômago, vergonha, medo, estresse e hipertensão arterial	As mulheres bolivianas vivenciam condições de trabalho precárias: jornadas superiores a 12 horas, pouco descanso e salários extremamente baixos, beirando a escravidão e a insalubridade, e sofrem racismo, classismo, machismo e xenofobia. Em relação à violência doméstica, por serem indocumentadas, sofrem com barreiras linguísticas, e o distanciamento do país de origem e a ausência de rede de apoio dificultam a busca por serviços de saúde. Algumas relatam ameaças de morte e violência financeira por parte dos parceiros
16. Supimpa et al. ^26^ (2023)	Mulheres venezuelanas, haitianas e tunisianas na cidade de Curitiba (Paraná)	Descrever as experiências de migrantes durante o trabalho de parto e o nascimento em duas maternidades públicas de Curitiba	Qualitativa − história oral temática híbrida e entrevistas semiestruturadas	Durante o processo de trabalho de parto e nascimento, as migrantes relataram dor, medo, solidão, tristeza, medo da cesariana e desconfiança da equipe de saúde. Houve também relatos de confiança nas equipes, felicidade e sensação de terem sido bem cuidadas pela equipe de enfermagem (cuidados com o recém-nascido, amamentação, higiene, etc.). Dificuldades de comunicação entre profissionais de saúde e migrantes também foram destacadas, revelando a necessidade de um cuidado intercultural sensível	Toques vaginais indesejados durante o parto e violência obstétrica
17. Silva & Justo ^27^ (2020)	Migrantes internas brasileiras (estados de São Paulo e Paraná)	Investigar a mobilidade de mulheres que transitam de cidade em cidade no interior do estado de Mato Grosso do Sul	Qualitativa − cartografia com entrevistas com duas mulheres	Pessoas em situação de rua são muitas vezes vistas socialmente como doentes, longe do ideal de família nuclear. Há relatos de ansiedade e choro infantil ao relembrar situações de violências, e relatos de uso de drogas lícitas e ilícitas e medicamentos	Mulheres migram para escapar da violência doméstica de gênero e buscar melhores condições de vida e trabalho. Uma entrevistada relatou ter sofrido violências física e emocional na infância devido à sua homossexualidade
18. Souza et al. ^28^ (2020)	Mulheres haitianas em diversas cidades brasileiras	Compreender as repercussões da COVID-19 e os determinantes sociais da saúde	Qualitativa − pesquisa-ação participativa com haitianas. Amostragem bola de neve (*snowball*)	Foram relatados medo da COVID-19, escassos recursos financeiros, impacto do racismo e do preconceito na saúde mental, dificuldades e sobrecarga em conciliar o trabalho e o cuidado da família durante a pandemia, uma vez que creches e escolas fecharam, desemprego, ansiedade, preocupação, estresse, choro e falta de sono. Os serviços de saúde e o apoio social fortalecem as estratégias de enfrentamento da pandemia	Há preconceito, racismo e discriminação no mercado de trabalho pelo fato de serem mulheres, recebendo salários inferiores aos dos homens
19. Gehlen et al. ^29^ (2023)	Mulheres venezuelanas no Rio Grande do Sul	Analisar as vulnerabilidades de mulheres venezuelanas e suas experiências de violência na condição de refugiadas	Qualitativa − entrevistas com venezuelanas residentes em um município do Rio Grande do Sul	A saúde mental se deteriorou (síndrome do pânico, depressão, sentimentos de tristeza, vergonha, humilhação e inferioridade) e doenças se agravaram com a migração; as relações familiares (com parentes e/ou parceiros) foram rompidas, com pouca ou nenhuma rede de apoio (familiar ou governamental). Relataram satisfação com os serviços do SUS; porém, sentiram que os profissionais de saúde tinham dificuldades em atendê-las	Xenofobia, fome, violência doméstica, racismo, machismo, violência sexual, estigmatização, classismo
20. Hora ^39^ ^)^ (2023)	Mulheres congolesas no Rio de Janeiro	Analisar o acesso de mulheres congolesas à atenção à saúde no Rio de Janeiro	Qualitativa − entrevistas com gestores e profissionais da ESF e mulheres congolesas	As congolesas afirmam que o atendimento nas clínicas da família é bom, mas identificam falta de suporte dentro do SUS (precarização do trabalho em saúde e subfinanciamento das políticas públicas no SUS). Há falta de mediação intercultural, atrasos no atendimento, assistência médica precária e demandas por planejamento familiar e pré-natal. Não há produção de dados sistematizados sobre a saúde das migrantes e há necessidade de formação profissional (educação permanente em saúde) no SUS, além da produção de materiais educativos sobre saúde para migrantes	A violência contra mulheres migrantes é um desafio, como no caso da violência sexual, e requer acompanhamento pelos serviços de saúde mental. Há violência obstétrica, racismo institucional, violência urbana e armada no Rio de Janeiro (tiroteios e operações policiais nos territórios onde vivem) e experiências de migração forçada
MULHERES BRASILEIRAS NO EXTERIOR					
21. Barata ^11^ (2022)	Mulheres brasileiras em Portugal	Discutir experiências de racismo e violência obstétrica contra mulheres brasileiras em Portugal	Qualitativa − entrevistas com três mulheres afro-brasileiras em Portugal, observação participante, grupos focais e criação artística participativa sobre o atendimento obstétrico	O fato de as mulheres relatarem sofrer violência obstétrica, violências xenofóbica e racial tem implicações para a saúde sexual e reprodutiva, autoestima e qualidade de vida, especialmente durante a gravidez e o parto. Uma mulher relatou depressão pós-parto associada à violência obstétrica. Barreiras de acesso aos serviços de saúde também são relatadas	Violência de gênero/obstétrica, manobra de Kristeller, abusos físicos e verbais, maus-tratos, objetificação do corpo, intervenções médicas não consensuais, episiotomias, procedimentos inadequados, xenofobia e racismo, hipersexualização das mulheres brasileiras e abusos verbais durante a amamentação em público foram constatados, entre outros incidentes. Além disso, era comum ouvir que as mulheres têm “úteros ruins” e são miscigenadas, o que reforça a xenofobia e o racismo
22. Chedid & Hemais ^30^ (2022)	Mulheres brasileiras no Norte Global	Analisar, por meio da teoria pós-colonial de Spivak, como mulheres brasileiras no contexto do turismo no Norte Global são subjugadas por estrangeiros	Qualitativa − estudo de caso através de 14 entrevistas com mulheres brasileiras que viajaram a turismo para o Norte Global	O artigo não aborda diretamente os resultados específicos em saúde. Entretanto, a subordinação aliada a experiências de violências física e psicológica podem ter impactos diretos na saúde das mulheres. A discriminação e a objetificação no contexto do turismo também podem causar sofrimento emocional significativo. Em relação à violência sexual, quando assediadas por homens, sentem-se amedrontadas, chateadas, ameaçadas, culpadas e incapazes de reagir imediatamente. A autonomia e o autocuidado reduzidos podem impactar sua capacidade de buscar assistência médica em ambientes estrangeiros	Os principais tipos de violência relatados são a psicológica e a física. A violência psicológica se expressa através de insultos e comentários sexistas e xenofóbicos, que produzem sentimentos de inferioridade. São tratadas como objetos de desejo e reforçam representações fetichizadas da nacionalidade brasileira. A violência física envolve abordagens agressivas, empurrões, toques indesejados e violência sexual, causando danos imediatos, além de medo e insegurança. Ademais, estão sujeitas ao machismo e à xenofobia
23. França & Oliveira ^12^ (2021)	Mulheres brasileiras em Portugal	Analisar as resistências expressas nas redes sociais por mulheres brasileiras em Portugal contra a discriminação e o preconceito	Qualitativa − entrevista com a coordenadora da rede social no Instagram “Brasileiras não se calam”, e análise do discurso das postagens e netnografia, sob perspectivas decoloniais e interseccionais	A partir das postagens feitas por mulheres brasileiras na rede social analisada, os sentimentos foram de frustração, tristeza e humilhação	Preconceito, discriminação, hipersexualização, machismo e racismo reiteram a colonialidade, ainda hoje, e seus impactos na vida das mulheres brasileiras no Norte Global, com relatos de violência simbólica, psicológica e moral, abusos verbais, assédio, e violência sexual. As mulheres brasileiras usam as redes sociais para denunciar as violências sofridas, destacando suas agências e ativismos
24. Gomes ^31^ (2013)	Mulheres brasileiras em Portugal	Analisar discursos preconceituosos e discriminatórios sobre mulheres brasileiras em Portugal	Qualitativa − observação participante, análise de discursos midiáticos e turísticos, uso de questionários e entrevistas	O artigo não se aprofunda muito em questões de saúde. No entanto, aponta que as mulheres brasileiras são estigmatizadas como aquelas que “trazem doenças” aos portugueses, especialmente as sexuais	Temos a hipersexualização e os discursos sobre o “corpo colonial”, que se ligam a estigmatizações como estarem sexualmente disponíveis e serem amantes de homens portugueses, assédio moral e sexual, violência física e sexual, e xenofobia. As trabalhadoras domésticas brasileiras sofrem assédio, especialmente sexual, racismo e machismo, além de atuarem em empregos precários
25. Mcllwaine & Evans ^32^ (2023)	Mulheres brasileiras em Londres (Inglaterra)	Analisar as violências de gênero contra mulheres brasileiras em Londres	Quantitativa/qualitativa - questionário, grupo focal e entrevistas com mulheres brasileiras e gestores de serviços de atendimento a migrantes	Sentimentos de vergonha, humilhação, medo (inclusive de deportação, se indocumentadas) e isolamento. As mulheres em situação de migração irregular tiveram mais dificuldade em acessar serviços públicos, como assistência social, segurança e saúde, bem como direitos trabalhistas. Uma migrante relatou depressão devido à violência por parceiro íntimo	Violência de gênero por parceiro íntimo, xenofobia, racismo, discriminação psicológica, física e sexual, e assédio sexual no local de trabalho. Casos de violência infraestrutural, ou seja, violência perpetrada por serviços que prestam atendimento a mulheres em situação de violência. A exploração das mulheres pela sociedade, o fato de estarem fora de seu país de origem e a violência estrutural são fatores que influenciam a decisão de manter relacionamentos com parceiros íntimos
26. Pelúcio ^33^ (2023)	Mulheres brasileiras no Japão, França, Irlanda, Itália, Canadá, Estados Unidos, Senegal, Inglaterra, Dinamarca, Noruega e Alemanha	Analisar as narrativas de mulheres brasileiras no exterior por meio do *podcast* Femigrantes BR	Qualitativa - análise de mídia social (*podcast*) sob a perspectiva dos feminismos negros	Um dos *podcasts* foi sobre saúde e interseccionalidade; no entanto, o artigo mal aborda esse tópico	Racismo, colonialidades do saber e do poder, machismo, sexismo, misoginia e mulheres brasileiras hipersexualizadas foram relatados. As mulheres negras veem sua trajetória educacional como uma das estratégias de enfrentamento às opressões do racismo

ESF: Estratégia Saúde da Família; SIM: Sistema de Informações sobre Mortalidade; SUS: Sistema Único de Saúde.

Fonte: elaborado pelas autoras.

## Aspectos gerais da amostra selecionada

Foram selecionados 26 documentos para análise: (a) 20 artigos ^11^ ^,^ ^12^ ^,^ ^16^ ^,^ ^17^ ^,^ ^18^ ^,^ ^19^ ^,^ ^20^ ^,^ ^21^ ^,^ ^22^ ^,^ ^23^ ^,^ ^24^ ^,^ ^25^ ^,^ ^26^ ^,^ ^27^ ^,^ ^28^ ^,^ ^29^ ^,^ ^30^ ^,^ ^31^ ^,^ ^32^ ^,^ ^33^; (b) dois livros ^34^ ^,^ ^35^; e (c) quatro teses/dissertações ^36^ ^,^ ^37^ ^,^ ^38^ ^,^ ^39^. Vinte documentos abordam mulheres migrantes no Brasil, dos quais: quatro tratam de migrantes de diversas nacionalidades ^20^ ^,^ ^21^ ^,^ ^26^ ^,^ ^36^; três de mulheres bolivianas ^24^ ^,^ ^25^ ^,^ ^37^; três de mulheres venezuelanas ^19^ ^,^ ^22^ ^,^ ^29^; dois de mulheres congolesas ^38^ ^,^ ^39^; um de uma migrante da Gâmbia ^17^; um de médicas cubanas ^23^; um de mulheres haitianas ^28^; e dois de migrantes internas brasileiras ^18^ ^,^ ^27^.

No que se refere à formação de profissionais de saúde ou à assistência a migrantes no Brasil, foram identificados dois documentos oficiais elaborados pelo Ministério da Saúde, publicados em 2012 ^34^ ^,^ ^35^. Além disso, foram incluídos seis artigos sobre mulheres brasileiras no exterior: (a) três sobre brasileiras em Portugal ^11^ ^,^ ^12^ ^,^ ^31^; (b) dois sobre brasileiras em diferentes países ^30^ ^,^ ^33^; e (c) um sobre brasileiras na Inglaterra ^32^.

A maioria dos estudos adota abordagem qualitativa, com uso de entrevistas, grupos focais e análise de literatura e de mídias sociais. Apenas um trabalho é quantitativos ^16^, e dois apresentam abordagem quali-quantitativa ^24^ ^,^ ^32^. Quanto à localização das migrantes mencionadas nas pesquisas no território brasileiro, identificaram-se: (a) quatro produções ^24^ ^,^ ^25^ ^,^ ^36^ ^,^ ^37^ realizadas na cidade de São Paulo; (b) três na cidade do Rio de Janeiro ^17^ ^,^ ^38^ ^,^ ^39^; (c) duas no Estado do Rio Grande do Sul ^18^ ^,^ ^29^; (d) duas no Estado de Roraima ^19^ ^,^ ^22^; (e) duas no Estado do Paraná ^21^ ^,^ ^26^; e (f) uma abrangendo os estados de São Paulo e Paraná ^27^. Como não foi estabelecido recorte temporal, observou-se que 18 documentos foram publicados entre 2020 e 2024 (cinco sobre brasileiras no exterior), enquanto oito produções foram publicadas entre 2012 e 2019, indicando o crescimento da temática da violência contra mulheres migrantes no Brasil e contra mulheres brasileiras no exterior nos últimos quatro anos.

## Saúde e migração: mulheres migrantes no Brasil e brasileiras no exterior

Esta seção apresenta uma síntese do que pode ser inferido da amostra quanto à relação entre saúde e migração, sendo abordadas as seguintes categorias: saúde mental, implicações das migrações forçadas forçada e das condições de trabalho para a saúde, impactos da violência na saúde, saúde sexual e reprodutiva e barreiras de acesso aos serviços de saúde.

Com relação à saúde das mulheres migrantes no país, observa-se que elas são heterogêneas e que essa condição está estritamente relacionada à nacionalidade, raça/etnia, geração, classe social, orientação sexual, tempo de permanência no país, proficiência na língua portuguesa, profissão e relações de trabalho (formal ou informal), além do *status* migratório. Nesse sentido, com base na ideia da pluralidade das mulheres e de suas condições de vida, considera-se que: “*Diferentes peculiaridades as singularizam e, com isso, demandam olhares específicos, tanto para análises acadêmicas quanto para seus possíveis desdobramentos em termos de elaboração de políticas públicas relacionadas a elas*” ^20^ (p. 3).

Os processos migratórios podem envolver dificuldades de adaptação, integração social, barreiras linguísticas e sociais e decisões que implicam deixar familiares e filhos no país de origem ^36^. Portanto, foi comum encontrar produções que complexificam os aspectos de saúde, relacionando-os às condições de vida, aos processos migratórios e às violências contra as migrantes.

Destacam-se as produções sobre a saúde mental das migrantes, uma vez que as discussões sobre sofrimento psicossocial e transtornos mentais se sobressaem na amostra ^19^ ^,^ ^21^ ^,^ ^34^ ^,^ ^35^ ^,^ ^38^. Quando os estudos dialogam com a interseccionalidade, a saúde mental aparece relacionada às opressões de raça, classe social e gênero, além das situações de vulnerabilização das migrantes, sendo o adoecimento expresso por sofrimento psicossocial, crises de ansiedade, distúrbios do sono, cefaleias, síndrome do pânico e necessidade de uso de psicotrópicos ^36^.

As mudanças nas condições de vida e nas redes de apoio social e familiar, as dificuldades com o idioma e a integração social, as tensões cotidianas, o isolamento, a irregularidade do processo migratório e a solidão vivenciada podem impactar a saúde, causando sofrimento e o desenvolvimento de transtornos mentais ^35^ ^,^ ^36^.

Os deslocamentos forçados agravam os obstáculos à manutenção e ao cuidado da saúde mental das migrantes: “*Quando nos referimos à migração forçada, estamos mencionando indivíduos que estão fugindo de guerras, perseguições políticas, religiosas, sociais ou violações de direitos humanos*” ^20^ ^)^ (p. 4). Em situações de refúgio, as mulheres tendem a desenvolver transtorno de estresse pós-traumático após deixarem seus países, sendo obrigadas a lidar com adaptações, vulnerabilização socioeconômica e violências ^20^.

Observam-se sofrimento social, solidão, ansiedade, esgotamento emocional, cefaleias, insônia, estresse, taquicardia, luto, depressão, desânimo, angústia, medos e traumas ^38^, além de tristeza ^29^ ^,^ ^38^, síndrome do pânico e sentimentos de vergonha, humilhação e inferioridade ^29^. A migração forçada e a perseguição política contra mulheres congolesas agravam o sofrimento e os transtornos mentais, reforçando a necessidade de cuidados em saúde mental ^39^. 

Arruda-Barbosa et al. ^19^ indicam que, no caso das mulheres venezuelanas, as condições de migração forçada, interseccionadas com opressões de raça e gênero, exacerbam o adoecimento mental e a vulnerabilidade às infecções sexualmente transmissíveis (ISTs), sobretudo quando exercem trabalho sexual. Em alguns casos, homens oferecem pagamento mais elevado para que não utilizem preservativos. Além disso, a vivência de violências está associada ao desenvolvimento de depressão e ansiedade. 

A migração forçada também é vivenciada por mulheres brasileiras que se deslocam internamente em busca de melhores condições de vida e trabalho, o que, aliado à violência estrutural, às condições precárias de moradia e à negligência estatal, tem implicações para sua saúde física e mental, exigindo a oferta de cuidados específicos e acessíveis ^18^. Mesmo quando o deslocamento ocorre voluntariamente, por exemplo para estudar dentro do Brasil, ou seja, fora do contexto de migração forçada, sofrimento psicológico, depressão, somatização, tristeza, baixa autoestima e ganho de peso podem ocorrer, especialmente quando se tornam mães, o que pode agravar sentimentos de medo e solidão e estar associado a transtornos de ansiedade ^21^.

Nesta revisão, o processo migratório está intimamente relacionado à saúde das mulheres que o vivenciam, com expressiva presença de sofrimento psicológico associado às condições de migração e de vida no destino ^16^ ^,^ ^34^. Dependendo do *status* migratório, como nos casos de documentação em situação irregular, elas enfrentam barreiras ainda maiores para acessar serviços de saúde, assistência social e outros, agravadas pela ausência de apoio social e familiar ^24^ ^,^ ^35^. 

A saúde das mulheres migrantes também está inter-relacionada a condições precárias de trabalho, exposição a riscos ocupacionais, insalubridade, violência laboral e tráfico de pessoas para trabalho escravo contemporâneo e/ou exploração sexual. Dependendo da localização, rural ou urbana, torna-se ainda mais difícil romper com redes de tráfico humano, o que agrava a vulnerabilidade psicológica e o adoecimento ^35^. É comum que essas mulheres estejam desempregadas ou inseridas no setor informal ^36^, o que compromete ainda mais o acesso às redes de cuidado.

As migrantes relatam rotinas exaustivas, com sobrecarga de trabalho doméstico e de cuidado familiar, que impede a fruição de tempo de lazer ^36^. Além disso, durante a pandemia de COVID-19, mulheres haitianas relataram dificuldades para conciliar o cuidado familiar com o trabalho, uma vez que muitas escolas e creches permaneceram fechadas, situação diretamente relacionada à sobrecarga do trabalho de cuidado ^28^.

Mulheres congolesas também relatam dificuldades para obter vagas em creches para seus filhos e o peso da divisão sexual do trabalho de cuidado. Além de serem responsáveis pelo cuidado, em alguns casos mencionam estar “a serviço” de seus parceiros, em conformidade com ensinamentos bíblicos cristãos. Ao mesmo tempo, consideram a subsistência econômica uma obrigação masculina, embora desejem encontrar emprego ^38^. Ainda no que se refere ao trabalho reprodutivo, mulheres venezuelanas são responsáveis pelo cuidado familiar e sentem-se obrigadas a desempenhá-lo ^22^.

Para aquelas que estão empregadas, as violências e as violações de direitos não são menos presentes. As mulheres bolivianas, por exemplo, são impedidas por seus empregadores de tirar licença-maternidade e de acessar os serviços de saúde, uma vez que qualquer tempo afastado das máquinas representa menos lucro para o capitalismo. Elas também estão distantes das redes de apoio e da família, o que dificulta a a saída das redes de exploração e das violências. Suas condições de trabalho frequentemente beiram a escravidão, com jornadas longas e intensas e salários irrisórios ^25^. 

Uma vez inseridas em oficinas de costura, elas podem deparar-se com condições de trabalho precárias, como a atuação em espaços mal ventilados, com mofo e problemas elétricos (frequentemente as mesmas condições de suas moradias), o que desencadeia problemas respiratórios e distúrbios do sono, bem como distúrbios musculoesqueléticos decorrentes de maquinário não ergonômico e sobrecarga de trabalho. Além de utilizarem seus salários para o próprio sustento e o de seus filhos, elas também enviam parte de sua renda para suas famílias na Bolívia ^37^.

O *status* migratório também pode ser utilizado como justificativa para baixos salários ^19^, exploração laboral, jornadas de trabalho intensas e moradias precárias ^21^. Soma-se a isso a divisão racial do trabalho, na qual mulheres negras ocupam posições de menor prestígio social, com salários ainda mais baixos e atividades mais precárias ^38^. 

Os artigos sobre mulheres brasileiras no exterior raramente exploram questões de saúde, sendo um dos pontos levantados as dificuldades de acesso a esses serviços ^11^ ^,^ ^32^. Quando se encontram em situação migratória irregular, enfrentam dificuldades para acessar serviços sociais, de saúde e de segurança, bem como para exercer seus direitos trabalhistas, sendo o medo da deportação uma realidade ^32^.

O medo é um sentimento recorrente nas pesquisas sobre mulheres brasileiras ^12^ ^,^ ^30^ ^,^ ^32^. Contudo, também se observam frustração, humilhação e tristeza ^12^, além de vergonha e culpa ^30^ ^,^ ^32^. Caso vivenciem violência obstétrica, xenofobia e racismo, as mulheres brasileiras podem desenvolver depressão pós-parto, o que impacta o autocuidado e a autoestima ^11^.

Outra questão levantada pelas mulheres brasileiras é a de que são socialmente consideradas como aquelas que “levam” doenças sexualmente transmissíveis para os homens estrangeiros − neste caso, os portugueses −, reiterando a estigmatização e a hipersexualização das mulheres dessa nacionalidade ^31^. Essa objetificação e discriminação podem causar estresse emocional e sentimentos negativos, como culpa e dificuldade de reagir à violência em situações de assédio perpetradas por homens ^30^. 

As barreiras de acesso aos serviços de saúde no Brasil foram identificadas reiteradamente como um fator social que compromete ainda mais a saúde das mulheres migrantes, as quais se encontram isoladas e privadas do direito ao cuidado ^35^. Oliveira ^36^ destaca a discriminação e a xenofobia perpetradas nos serviços de saúde. O uso de expressões xenofóbicas por profissionais durante o parto e o pós-parto também foi identificado ^20^ ^,^ ^37^, assim como barreiras culturais e linguísticas ^37^ ^,^ ^39^. Tais realidades distanciam ainda mais as mulheres do seu direito à saúde e constituem motivos para a não busca por atendimento em situações de violência doméstica ^25^.

No que tange às abordagens linguísticas e culturais em saúde, ressalta-se a relevância e a urgência de ofertar programas de mediação intercultural para que as migrantes possam receber atendimento de qualidade e ter seu direito à saúde garantido ^39^. Deve-se lembrar que um dos motivos para a vinda ao Brasil é a possibilidade de utilização dos serviços do Sistema Único de Saúde (SUS), a exemplo da assistência à gravidez e à maternidade, consultas com endocrinologistas e ginecologistas e vacinação ^36^. Portanto, assegurar um cuidado que considere as dimensões interculturais é fundamental para a prestação adequada da assistência em saúde.

As publicações voltadas à formação de trabalhadoras no Brasil partem desse reconhecimento para construir possibilidades de acesso e cuidado ^34^ ^,^ ^35^, considerando a responsabilidade do poder público no enfrentamento das violências contra migrantes, notadamente o tráfico de pessoas nas modalidades de trabalho escravo contemporâneo, exploração sexual, casamento servil e tráfico de órgãos. Essa perspectiva fundamenta o desenvolvimento de propostas de formação para profissionais da rede SUS, que devem ser sensíveis às vulnerabilizações implícitas na exposição a “*riscos de contágios, infecções, adoecimentos, sofrimentos psíquicos e transtornos mentais*” ^35^ (p. 35). O risco significativo para a saúde sexual e reprodutiva ^35^, bem como a dificuldade de acesso aos serviços que ofertam esse cuidado, é destacado e reconhecido como uma realidade no país ^34^. Há necessidade de que os serviços identifiquem e intervenham em situações de violência contra mulheres migrantes ^34^, reconhecendo que muitas podem sofrer violência institucional nos serviços de saúde ^35^ ^,^ ^36^.

No que se refere às estratégias de cuidado em saúde mental para mulheres migrantes, encontra-se uma menção importante à criação de um campo específico de produção de conhecimento e cuidado, denominado “*‘psicologia do migrante’; ‘antropsiquiatria’; ‘psicologia transcultura’; ‘psicoterapia transcultural’*” ^35^ (p. 49), a fim de compreender os efeitos da migração na saúde mental, no sofrimento e no adoecimento ^35^.

A “psicologia da migração” tem se concentrado na análise de fatores macrossociais, carecendo de maior atenção a fatores mais próximos ao indivíduo implicados no adoecimento, tais como aspectos subjetivos, comportamentais, socioculturais e intersubjetivos ^35^. Contudo, critica-se a perspectiva medicalizante que reduz o sofrimento ao indivíduo e que frequentemente orienta as práticas de cuidado em saúde mental.

## Violência e migração: mulheres migrantes no Brasil e brasileiras no exterior

Esta seção apresenta os tipos de violência contra migrantes identificados na amostra, incluindo violência de gênero, racial, xenofóbica e institucional. A migração forçada é compreendida aqui como um fenômeno violento que vulnerabiliza as mulheres ao obrigá-las a se deslocarem de seus locais de origem devido às violências direta, como as violências baseadas em gênero, e às violências indiretas, como a violência estrutural, que torna determinados territórios insustentáveis para a vida − a exemplo da fome, da presença de grupos armados e de desastres climáticos, entre outros fatores ^40^. 

Ressalta-se que, embora alguns documentos façam referência específica a determinados tipos de violência, uma ampla gama de produções ^11^ ^,^ ^12^ ^,^ ^19^ ^,^ ^25^ ^,^ ^29^ ^,^ ^31^ ^,^ ^32^ ^,^ ^33^ ^,^ ^36^ ^,^ ^37^ ^,^ ^38^ ^,^ ^39^ aborda a violência contra migrantes de forma interseccional, evidenciando a complexidade das opressões em seu cotidiano.

A pesquisa de Barata ^11^, por exemplo, intersecciona e racializa a violência obstétrica, contextualizando as relações coloniais que marcaram o Brasil e Portugal. Oliveira ^36^, baseando-se em Kimberlé Crenshaw e Carla Akotirene, expõe as opressões de raça, classe social, gênero e nacionalidade, demonstrando que as violências coloniais − como o racismo, a xenofobia e a desigualdades de gênero − influenciam as condições de vida de migrantes e refugiadas. Hora ^39^ mostra que a violência sexual, obstétrica e armada nos grandes centros urbanos, além do racismo institucional, podem impactar a saúde mental das mulheres congolesas.

Diante do exposto, empreendeu-se um esforço para organizar os tipos de violência segundo sua natureza e suas expressões, a fim de compor um quadro capaz de visualizar a realidade social das mulheres migrantes. No entanto, a intersecções dos tipos de violência descritos em cada artigo ilustram claramente a complexa submissão a estruturas de poder xenofóbicas, misóginas, racistas e classistas, aproximando esse quadro de uma miríade caleidoscópica. Assim, destaca-se que, apesar do movimento em direção à síntese dos estudos, essa perspectiva da complexidade intrincada da violência deve ser considerada.

A fome, enquanto expressão da violência estrutural, é uma realidade para milhões de mulheres e famílias. A amostra identificou que as migrantes vivenciam violências intrinsecamente ligadas à produção de vulnerabilidade ^19^ ^,^ ^20^ ^,^ ^36^: “*Faz dois dias que eu não sei o que é comer, tanto por causa da dor quanto pela falta de dinheiro. Às vezes eu penso que se tivesse uma arma já teria ido embora, não aguento mais sofrer assim*” ^19^ (p. 4). Esse excerto também indica a inter-relação entre a fome e a fragilidade da saúde mental, culminando em uma situação grave na qual a mulher entrevistada relatou ideação suicida. 

Outra questão levantada, embora raramente, refere-se à relação direta entre as violências e a saúde das migrantes. Arruda-Barbosa et al. ^19^ argumentam que a exploração sexual contra mulheres venezuelanas está associada ao sofrimento psicológico, à depressão e à ansiedade. Migrantes que vivenciaram ou vivenciam violência doméstica relatam temer seus parceiros e, quando acolhidas em abrigos, também temem outros homens com quem compartilham o espaço, especialmente quando precisam utilizar os banheiros do local ^22^. A violência doméstica também está relacionada à depressão, dores de estômago, vergonha, medo, estresse e hipertensão arterial ^25^. De modo semelhante, a depressão relaciona-se a situações de violências por parceiro íntimo ^32^. 

A subnotificação de casos de violências e feminicídio, bem como falhas no registro de dados, como no campo raça/cor, também configuram um problema grave, especialmente em municípios de pequeno porte, municípios fronteiriços e naqueles com populações indígenas ^16^. Somam-se a isso as subnotificações de violências contra mulheres migrantes, sobretudo daquelas em situação de tráfico e exploração sexual e em situação migratória irregular, dado que frequentemente se encontram sob o controle de redes criminosas internacionais ^34^. 

O tráfico de pessoas para trabalho escravo contemporâneo (TEC), a exploração sexual e a prostituição forçada ocorrem nas regiões de fronteira do Brasil ^16^ ^,^ ^35^, com mulheres bolivianas inseridas no TEC ^37^, onde as mulheres mais pobres, desempregadas, negras e jovens são as mais expostas a essas violências ^35^. Em situações de exploração sexual, a condição de refugiada pode intensificar o racismo, a xenofobia, a misoginia e a pobreza ^19^.

Diversos tipos de violência baseada em gênero foram relatados na amostra, como violência sexual ^16^ ^,^ ^20^ ^,^ ^21^ ^,^ ^24^ ^,^ ^29^ ^,^ ^39^, estupro ^19^ ^,^ ^24^, ocorrido no local de trabalho ^35^ e no contexto de violência armada no país de origem ^38^; violência psicológica ^20^ ^,^ ^22^ ^,^ ^35^; violência obstétrica ^26^ ^,^ ^37^ ^,^ ^39^; casamento infantil no país de origem como forma de submissão ao patriarcado e às desigualdades de gênero ^17^; violências nas relações domésticas ^18^ ^,^ ^22^ ^,^ ^29^ ^,^ ^38^, com abusos físicos e verbais e ameaças psicológicas ^22^; ameaças com o uso de armas brancas ^19^; e violência física ^24^ ^,^ ^35^ nas relações domésticas, conforme mencionado, também associada ao trabalho sexual ^19^. As desigualdades de gênero entre estudantes universitárias também foram objeto de um estudo ^21^.

Além das tipologias de violência já descritas, identificam-se o racismo interpessoal contra mulheres negras ou indígenas ^21^ ^,^ ^29^ ^,^ ^36^ ^,^ ^37^ ^,^ ^39^, o racismo institucional nos serviços de saúde ^38^ e o racismo contra profissionais de saúde cubanas ^23^. Neste último caso, o racismo manifesta-se quando mulheres brasileiras, nas redes sociais, questionam a profissão dessas profissionais − seriam realmente médicas? − e as tratam como trabalhadoras domésticas ^23^. Embora não mencionado explicitamente no artigo, observa-se também a misoginia interseccionada a essas violências.

As relações entre racismo, xenofobia e saúde mental são destacadas em três documentos ^28^ ^,^ ^38^ ^,^ ^39^. Em síntese: “*Sobre a saúde mental das refugiadas entendeu-se que o sofrimento mental não se restringe a fatores meramente psicopatológicos ou apenas biomédicos, ele ocorre por causas sociais como a precariedade, as injustiças, as perdas, a separação familiar, a xenofobia, o racismo e a violência*” ^39^ (p. 194).

Diante do exposto, observa-se que diversas formas de violência contra mulheres migrantes se interseccionam em alguns estudos, comprometendo a saúde mental dessas mulheres. Especificamente, a xenofobia é apontada como um tema relevante na amostra ^17^ ^,^ ^19^ ^,^ ^20^ ^,^ ^22^ ^,^ ^28^ ^,^ ^29^ ^,^ ^35^ ^,^ ^36^ ^,^ ^37^, sendo vivenciada diretamente pelas mulheres migrantes e, em alguns casos, por suas filhas e filhos ^37^ ^,^ ^38^.

A perseguição política no país de origem é tratada na literatura ^36^ ^,^ ^38^ como uma das possíveis motivações para a saída da terra natal, assim como a violência estrutural ^18^ ^,^ ^21^, que leva ao deslocamento forçado para outros países em busca de melhores condições de vida ^20^. As violências também se configuram como motivação para o deslocamento, como no caso de uma mulher que deixou seu lar após sofrer violências física e moral na infância em razão de sua homossexualidade ^27^.

A violência patrimonial no local de trabalho ^19^ ^,^ ^37^, em abrigos para refugiados ^22^ e por parte de parceiros íntimos ^25^ também afeta as mulheres, assim como as ameaças de deportação ^37^. O Estado brasileiro negligencia a migração forçada decorrente de questões ambientais, o que gera precarização nas condições de vida de mulheres que enfrentam desterritorialização, endividamento e envolvimento com o narcotráfico ^18^, além da violência armada em grandes centros urbanos, como o Rio de Janeiro ^39^. Destaca-se que apenas um estudo mais recente evidencia também discriminações expressas por meio do capacitismo, do etarismo contra migrantes idosas, da intolerância religiosa, do racismo, do sexismo e da heteronormatividade ^20^.

Os artigos que apresentam resultados de estudos sobre mulheres brasileiras no exterior contextualizam e historicizam a colonialidade e as representações das mulheres latino-americanas, especialmente em cinco artigos ^11^ ^,^ ^12^ ^,^ ^30^ ^,^ ^31^ ^,^ ^33^, ressaltando a importância desse contexto para a compreensão dos discursos perversos e violentos em torno dos corpos das mulheres brasileiras: “*O problema está na própria existência do estigma de prostituta, o qual, relacionado com a colonialidade, o sexismo e o racismo, cria papéis e imaginários para as mulheres, sendo as brasileiras consideradas as ‘pecadoras, Evas, prostitutas, disponíveis, inferiores, hipersexualizadas’. O problema está, portanto, no estigma de ‘corpo colonial’, corpo disponível, que atinge todas as brasileiras*” ^31^ (p. 880).

Mulheres brasileiras no exterior também sofrem violências baseadas em gênero, como a violência obstétrica, marcada por diversos procedimentos invasivos e não consensuais ^11^. Nesse contexto, há igualmente relatos de racismo e xenofobia no cotidiano ^11^ ^,^ ^30^ ^,^ ^31^ ^,^ ^33^ e durante a gestação e o parto. As expressões de violência de gênero vivenciadas por brasileiras incluem fetichização, hipersexualização e objetificação, conforme discutido anteriormente ^12^ ^,^ ^30^ ^,^ ^31^ ^,^ ^33^. A violência sexual também é destacada e vinculada a questões estruturais, como o machismo e a xenofobia, que ampliam medos e inseguranças ^30^.

Apesar das opressões e violências, os estudos também ressaltam a resistência e o protagonismo das migrantes ao longo de suas trajetórias. São desenvolvidas estratégias para obtenção de emprego e para o cuidado próprio e familiar, com o objetivo de garantir direitos. Observa-se também atuação diante do racismo, explicitando seu caráter discriminatório aos perpetradores, além do reconhecimento da divisão racial do trabalho ^38^. No caso de mulheres brasileiras em Portugal, há enfrentamento de resistências e promoção de pautas por meio de movimentos sociais em rede, com apoio e participação de outras migrantes e de mulheres portuguesas ^31^.

## Discussão

“*Vocês partiram o mundo*

*em vários pedaços e*

*chamaram de países*

*declararam posse sobre*

*o que nunca lhes pertenceu*

*e deixaram os outros sem nada*

*− colonizado*” 

Rupi Kaur ^41^ (p. 137).

As palavras contundentes de Rupi Kaur ^41^ ecoam nesta revisão de literatura e associam-se aos percursos migratórios de mulheres migrantes no Brasil e de brasileiras no exterior. Evidencia-se que tais mulheres enfrentam a materialização de processos históricos de invasão e colonização que intensificaram inúmeras formas de violências contra seus corpos. Verifica-se que as violências contra migrantes estão circunscritas pela colonialidade de gênero, na qual dominação, relações de poder e o mandato das masculinidades estruturam abusos, violência psicológica, estupro e feminicídio ^6^. É também no interior do padrão colonial de gênero que “(...) *as mulheres e suas proles se tornam vulneráveis e matáveis como nunca antes*” ^6^ (p. 24). 

A revisão demonstra que as violências associada aos processos migratórios representam adversidades enfrentadas por mulheres latino-americanas. Contudo, simboliza também a construção de resistências traduzidas em ações de enfrentamento ao racismo, à xenofobia, ao sexismo, à violência baseada em gênero e à divisão sexual e racial do trabalho, nem sempre acompanhadas por redes de apoio ou por ações estatais voltadas à garantia de direitos. A migração forçada, que engloba pessoas refugiadas, deslocadas internas, solicitantes de asilo, apátridas e migrantes de crise e sobrevivência, caracteriza-se por intensas violações de direitos humanos e por violências contra mulheres ^42^. Esse processo não se encerra na chegada ao Brasil como país de destino, onde persistem diversas situações de violência com implicações para vida, saúde e trabalho.

Embora alguns estudos enfoquem o *status* de refúgio e sua relação com violência e saúde ^19^ ^,^ ^20^ ^,^ ^29^ ^,^ ^36^ ^,^ ^38^ ^,^ ^39^, observa-se que as migrações forçadas emergem articuladas às violências, sobretudo quando são motivadas a deixa a terra natal porque são violentadas. 

No entanto, destaca-se o fato de que, independentemente do *status* migratório − inclusive entre aquelas que chegam ao Brasil para fins de estudo ^21^ −, as violências permanecem recorrentes, o que reafirma a insuficiência de uma única categoria analítica para compreender suas múltiplas expressões. Por seu caráter interseccional e complexo, tais violências não podem ser padronizadas, devendo-se considerar perspectivas históricas e intersecções de opressões relacionadas à nacionalidade, classe social, raça/etnia, *status* migratório, entre outras ^3^ ^,^ ^7^ ^,^ ^9^.

Ainda que muitos estudos não explicitem ou conceituem a interseccionalidade, parte da amostra ^11^ ^,^ ^12^ ^,^ ^19^ ^,^ ^24^ ^,^ ^25^ ^,^ ^29^ ^,^ ^31^ ^,^ ^32^ ^,^ ^33^ ^,^ ^36^ ^,^ ^37^ ^,^ ^38^ ^,^ ^39^ evidencia composições de expressões de violência articuladas a iniquidades estruturais, tanto no país de origem quanto no Brasil. Nessa perspectiva, a interseccionalidade, como ferramenta analítica, teórica e metodológica ^7^, mostra-se fundamental para pesquisas sobre mulheres migrantes, inclusive para a formulação de políticas públicas eficazes que as reconheçam como protagonistas de seus deslocamentos ^9^. 

Os estudos indicam intersecções entre condições de vida, trabalho produtivo − marcado por exploração, precariedade, insalubridade e baixa remuneração − e condições reprodutivas, caracterizadas pela sobrecarga de trabalho doméstico e de cuidado, com repercussões na saúde mental das mulheres migrantes. Observa-se acúmulo de sofrimento, sintomas e adoecimentos, agravados pela intersecção de diferentes formas de violências. Identificam-se casos extremos em que o desejo de pôr fim à própria vida surge como tentativa de interromper o sofrimento ^19^, inclusive em contextos nos quais o feminicídio não ocorre previamente ^16^.

No que se refere ao trabalho, a revisão aponta experiências que se aproximam de condições análogas à escravidão contemporânea e à exploração sexual, com impactos no adoecimento físico e mental decorrente da exploração laboral e da violência no ambiente de trabalho. Socialmente responsabilizadas pelo cuidado, muitas mulheres acumulam a criação dos filhos e o sustento familiar sem redes de apoio ou suporte estatal, chegando a criar os filhos em ambientes de trabalho, como ocorre especialmente em oficinas de costura ^43^, onde espaço laboral e vida familiar se imbricam. 

Ao correlacionar sofrimento e adoecimento mental às condições de vida, os estudos adotam uma perspectiva desindividualizada da saúde mental, compreendida não como atributo exclusivamente pessoal, mas como socialmente determinada. Condições de moradia, trabalho, alimentação, circulação, proteção, documentação, acesso a serviços e exposição a violências − como racismo e xenofobia − compõem o quadro de determinação social do processo saúde-doença, contribuindo para a fragilização da saúde física e mental de mulheres migrantes.

Considerando-se o lugar subjetivo e material ocupado por mulheres majoritariamente latino-americanas que buscam proteção e melhores condições de vida, impõe-se o peso da herança colonial que as insere em uma matriz de poder produtora e reprodutora de desigualdades sociais e de restrições quanto aos espaços que lhes são permitidos ocupar, sobretudo quando acumulam marcas de subalternização ^44^. Assim, raça, gênero, sexualidade, classe social e nacionalidade operam como marcadores de hierarquização que ampliam vulnerabilizações à medida que tais mulheres se distanciam do modelo hegemônico de indivíduo, especialmente em sociedades estruturadas por bases racistas, sexistas, patriarcais e xenofóbicas.

Segundo Lélia Gonzalez ^45^, tais marcas constituem elementos estruturantes da formação social brasileira, incidindo sobre todas as esferas da vida individual e coletiva e produzindo efeitos diretos e indiretos que naturalizam estereótipos e justificam a exploração sexual, o trabalho insalubre e mal remunerado e a sobrecarga do trabalho reprodutivo, particularmente entre mulheres negras.

Reconhecendo-se que relações sociais e formas capitalistas de matriz colonial influenciam as condições de vida das migrantes ^9^ ^,^ ^46^, torna-se inviável homogeneizar suas experiências ou desconsiderar as contradições entre avanços e retrocessos em seus deslocamentos. No campo da saúde mental, além das experiências inerentes aos processos migratórios, fatores econômicos, ausência de documentação e fragilidade de apoio social e familiar podem relacionar-se às demandas por cuidado. Tais dimensões nem sempre são adequadamente compreendidas por serviços e profissionais de saúde, que por vezes adotam perspectivas patologizantes diante de trajetórias plurais e singulares ^46^.

As experiências de migrantes com os serviços de saúde no Brasil revelam contradições significativas. Embora alguns estudos destaquem o Sistema Único de Saúde (SUS) como via relevante de acesso ao direito à saúde, identificam-se múltiplas barreiras. O acesso e a permanência nas redes de cuidado são condicionados por barreiras linguísticas, violência institucional, racismo e xenofobia presentes nos próprios serviços.

Evidencia-se, portanto, a necessidade de formação específica de profissionais para o atendimento a migrantes, conforme previsto nas diretrizes do SUS. Uma nota técnica recente do Ministério da Saúde referente ao cuidado de migrantes na atenção primária à saúde (APS) estabelece que a garantia da assistência requer respeito à cultura e ao idioma, atendimento independentemente da apresentação de documentos pessoais e disponibilização de materiais em múltiplos idiomas, entre outros princípios ^47^.

Práticas de saúde culturalmente sensíveis demandam o reconhecimento e a articulação da perspectiva teórico-metodológica da interseccionalidade ^7^ para ampliar a compreensão dos processos de saúde e adoecimento. No campo da saúde mental, tal abordagem permite superar determinismos reducionistas que responsabilizam indivíduos de forma isolada, incorporando fatores macro, meso e micropolíticos socialmente (re)produzidos. Também oferece subsídios relevantes para políticas públicas fundamentadas no reconhecimento e na assunção de responsabilidade pela formação de serviços e profissionais atentos, sensíveis, acolhedores e mais eficazes no cuidado e atenção às mulheres migrantes, com vistas ao enfrentamento das violências e à proteção de sua saúde física e mental.

Dada a complexidade das relações sociais e históricas que envolvem as migrantes, a proposta de cuidado intercultural no campo da saúde valoriza a reflexão sobre as práticas e os processos socioculturais existentes entre pessoas e grupos, reconhecendo suas heterogeneidades e as influências das desigualdades sociais, do racismo e da violência estrutural na atenção à saúde. É fundamental que as políticas públicas de saúde assegurem maior participação popular para sua efetiva implementação ^46^ ^,^ ^48^. 

Com base nesta revisão de literatura, recomenda-se maior aprofundamento teórico e metodológico acerca dos agenciamentos das migrantes, que, dia após dia, em seus movimentos sociais, buscam o direito à saúde e ao trabalho, livres de violência e convictas de que são protagonistas de suas próprias histórias, conforme apontam pesquisas sobre interseccionalidade e feminismos negros e/ou decoloniais ^6^ ^,^ ^7^ ^,^ ^9^ ^,^ ^45^.

Outra abordagem apresentada na revisão refere-se às mulheres brasileiras no exterior. Sob uma perspectiva feminista, destaca-se que a maioria dos artigos aborda a fetichização e a hipersexualização das mulheres brasileiras no Norte Global. Estar distante do país de origem, inserida em outra cultura e vivendo em um território onde se forjou a colonialidade de gênero ^6^ faz emergir reflexões sobre o sofrimento e os desafios enfrentados na luta cotidiana por melhores condições de vida para si e para suas famílias. Além disso, esse contexto levanta questionamentos acerca das políticas de saúde pública a elas destinadas: quais são essas políticas? Quem é responsável pela prestação de serviços de saúde, considerando sua condição de migrantes? Por outro lado, observa-se que seus movimentos de resistências ecoam globalmente: “*Suas vozes falam de experiências de serem mulheres expatriadas, enovelam-se com estruturas macrossociais e conectam-se a lutas pretéritas, revisitadas a partir de uma outra ótica feministas, aquela que tem colocado o mundo de cabeça para baixo*” ^33^ (p. 23).

Apesar dos contextos heterogêneos do Brasil e dos países do Norte Global para os quais as brasileiras migram, uma diferença relevante reside no fato de que nem sempre há acesso a serviços públicos universais de saúde, como ocorre com o SUS, que, apesar de seus desafios, atende parte da população migrante no país. Entre as semelhanças, a violência destaca-se de forma contundente, evidenciando a necessidade de cuidado ao longo de todo o percurso migratório.

Uma limitação deste estudo consiste na ausência de análise da qualidade metodológica das pesquisas incluídas. A escassez de literatura sobre mulheres brasileiras no exterior também restringiu o alcance para além de Portugal e Inglaterra. Esse percurso, contudo, pode ser explorado em investigações futuras, ampliando-se a atenção à violência que afeta mulheres brasileiras, especialmente quando buscam novas oportunidades no Norte Global. 

## Considerações finais 

Os estudos analisados evidenciam a violência interseccional que incide sobre mulheres migrantes no Brasil e mulheres brasileiras no exterior, reconhecendo-se a impossibilidade de generalizações diante das diversidades de trajetórias e experiências. Ainda assim, verifica-se que as violências se relacionam com o gênero, raça, classe social, nacionalidade, geração, deficiência e *status* migratório, conforme indicado por pesquisas interseccionais que destacam a complexa interação de opressões responsáveis por suas vulnerabilizações.

No que se refere aos processos de saúde e adoecimento, observa-se que condições de vida e trabalho, segurança alimentar, moradia, mobilidade e acesso — ou sua ausência — aos serviços de saúde e assistência social, bem como situações de violências (racismo, xenofobia, violências baseadas em gênero, etarismo, perseguição política, capacitismo, entre outras), estão inter-relacionadas aos adoecimentos físicos e mentais. Tais fatores, em muitas ocasiões, repercutem no bem-estar e no cuidado de si e da família.

A dinâmica do trabalho familiar, historicamente atribuída às mulheres, especialmente às mulheres negras e indígenas, constituiu uma das abordagens destacadas na revisão. Portanto, o trabalho não remunerado ou sub-remunerado desempenhado por mulheres deve ser considerado nas análises sobre a saúde de mulheres migrantes, uma vez que a sobrecarga de atividades laborais é recorrente. Apesar dos desafios impostos por sistemas de opressões e violências, a revisão evidencia práticas de resistências e protagonismos das migrantes ao longo de suas jornadas. Tais estratégias são projetadas tendo em vista a garantia de direitos enquanto migrantes e mulheres. Nesse sentido, elas não se encontram sozinhas. Por meio de movimentos coletivos, enfrentam e reafirmam as resistências às violências coloniais em um mundo onde “*declararam posse sobre o que nunca lhes pertenceu e deixaram os outros sem nada*”, conforme dito por Rupi Kaur ^41^ (p. 137).

## Data Availability

Os bancos de dados utilizados no estudo, incluindo os códigos de extração, análises e resultados estão disponíveis em repositório: (https://osf.io/c9vba/overview).
